# Impact and centrality of attention dysregulation on cognition, anxiety, and low mood in adolescents

**DOI:** 10.1038/s41598-023-34399-y

**Published:** 2023-06-05

**Authors:** Clark Roberts, Barbara J. Sahakian, Shuquan Chen, Samantha N. Sallie, Clare Walker, Simon R. White, Jochen Weber, Nikolina Skandali, Trevor W. Robbins, Graham K. Murray

**Affiliations:** 1 Department of Psychiatry, University of Cambridge, Cambridge, UK; 2 Department of Psychology, University of Cambridge, Cambridge, UK; 3 Behavioural and Clinical Neuroscience Institute, University of Cambridge, Cambridge, UK; 4 Department of Counseling and Clinical Psychology, Columbia University Teachers College, New York, NY USA; 5 University of Cape Town, Cape Town, South Africa; 6 MRC Biostatistics Unit, University of Cambridge, Cambridge, UK; 7 Memorial Sloan Kettering Cancer Center, New York, NY USA; 8 Cambridgeshire and Peterborough NHS Foundation Trust, Cambridge, UK

**Keywords:** Psychology, Human behaviour

## Abstract

Functional impairments in cognition are frequently thought to be a feature of individuals with depression or anxiety. However, documented impairments are both broad and inconsistent, with little known about when they emerge, whether they are causes or effects of affective symptoms, or whether specific cognitive systems are implicated. Here, we show, in the adolescent ABCD cohort (N = 11,876), that attention dysregulation is a robust factor underlying wide-ranging cognitive task impairments seen in adolescents with moderate to severe anxiety or low mood. We stratified individuals high in DSM-oriented depression or anxiety symptomology, and low in attention deficit hyperactivity disorder (ADHD), as well as vice versa – demonstrating that those high in depression or anxiety dimensions but low in ADHD symptoms not only exhibited normal task performance across several commonly studied cognitive paradigms, but out-performed controls in several domains, as well as in those low in both dimensions. Similarly, we showed that there were no associations between psychopathological dimensions and performance on an extensive cognitive battery after controlling for attention dysregulation. Further, corroborating previous research, the co-occurrence of attention dysregulation was associated with a wide range of other adverse outcomes, psychopathological features, and executive functioning (EF) impairments. To assess how attention dysregulation relates to and generates diverse psychopathology, we performed confirmatory and exploratory network analysis with different analytic approaches using Gaussian Graphical Models and Directed Acyclic Graphs to examine interactions between ADHD, anxiety, low mood, oppositional defiant disorder (ODD), social relationships, and cognition. Confirmatory centrality analysis indicated that features of attention dysregulation were indeed central and robustly connected to a wide range of psychopathological traits across different categories, scales, and time points. Exploratory network analysis indicated potentially important bridging traits and socioenvironmental influences in the relationships between ADHD symptoms and mood/anxiety disorders. Trait perfectionism was uniquely associated with both better cognitive performance and broad psychopathological dimensions. This work suggests that attentional dysregulation may moderate the breadth of EF, fluid, and crystalized cognitive task outcomes seen in adolescents with anxiety and low mood, and may be central to disparate pathological features, and thus a target for attenuating wide-ranging negative developmental outcomes.

## Introduction

Impaired performance in cognitive tasks appears to be a transdiagnostic feature of psychopathology (C-factor)^1^, and factor models indicate a large degree of covariation among all forms of psychopathology in population samples (P-factor)^2^. However, the precise causal relationships between a putative C-factor and a P-factor are unclear. Further, while the fitting of such models may provide insight into transdiagnostic features of psychopathology and cognitive impairments, they do little to explain the specific psychological mechanisms or symptoms in question^3,4^. Therefore, our primary objective was to assess if attention dysregulation may be a critical component of transdiagnostic cognitive deficits and disparate psychopathological features. This would have important implications for future research examining cognitive deficits in psychopathology and potential clinical impact.

Mapping specific experimentally identifiable cognitive dysfunctions to particular categorical disorders has been largely disappointing as nearly all clinical disorders show similar general deficits^1,5^. Nevertheless, attention dysregulation appears to be a feature common in most psychopathologies, but whether it is a cause or effect, or both, is still unclear. Although attention is notoriously difficult to delineate as a unitary construct^6^, attention dysregulation can refer to inefficient regulation or control of self-motivational systems directing normative top-down goal-directed action. Attention dysregulation is a paradigmatic characteristic of ADHD^7^, a disorder which usually precedes clinical anxiety and mood disorders in development^8^ and appears to increase their future likelihood of occurring^9,10^. Core features of ADHD disrupt functioning in many contemporary educational, vocational, and other social domains^11^, providing several straightforward connections to the pathogenesis of low mood and anxiety.

However, in contrast, phases of low mood and anxiety may still reflect intact control systems^12^ that are temporarily weighted towards processing aversive information and top-down predictions^13^, making it difficult to determine whether their frequently associated cognitive impairments are a cause or effect^1^. Dimensions of perception, affect, behaviour, and cognition functionally shift in tandem^13^, which might explain some of the transient and inconsistent cognitive impairments induced by low mood or anxiety.

We firstly hypothesized that the dimension of attention dysregulation (measured here by metrics of concentration problems or distractedness derived from parental ratings), whether as a cause or effect of psychopathology, would largely account for many transdiagnostic cognitive deficits (particularly among mood and anxiety disorders). This was based on the reasoning that more robust attentional regulatory control should allow for better one-shot learning, more efficient task sampling and goal-directed action, sequential reasoning in complex tasks, and perhaps motivating task interest. To test this first hypothesis, we (1) examined associations between ranging dimensions of psychopathology and cognitive performance across a range of tasks controlling for attentional problems and (2) stratified individuals who were high in ADHD but low in depression/anxiety, and vice versa*—*for a clearer examination of potential general cognitive deficits which might be dependent on attention dysregulation as opposed to composite scores of depression or anxiety symptomology.

Our second hypothesis (presented sequentially in Results) concerned the key role for attention dysregulation that would be manifest in network analysis of psychopathology. We hypothesized that attention dysregulation would exacerbate diverse psychopathological dimensions, have high centrality via its expected influence (sign of all positive and negative edge weights with other dimensions)^14,15^, and possibly exert a hierarchical influence on other dimensions^16^. Our reasoning was that features of ADHD are already well known to overlap widely and influence other disorders^10,17^, and attention dysregulation specifically conceivably increases general susceptibility towards aversive state changes. Therefore, in a confirmatory and synergistic manner, we examined the centrality of attention dysregulation and its hierarchical influence with other psychopathological features of anxiety, low mood, oppositional defiant disorder, and negative peer relations. Finally, exploratory analysis was also used to examine close relationships between higher-dimensional features and possible bridging connections.

## Methods

We studied the longitudinal Adolescent Brain Cognitive Development (ABCD) cohort (11,876 adolescents aged 9-12), which has an extensive cognitive task battery, a considerable sample size, and wide-ranging measures of psychopathology, to determine whether there was evidence for our hypotheses.

### Mental health outcomes

Dimensional mental health ratings were extracted from the previously validated Parent–Child Behavioral Checklist (CBL)^18–20^ which contains distinct DSM-oriented symptoms and sum scores related to ADHD, anxiety, depression, and other dimensions. Parents rated these measures on how frequent and accurate they were regarding their child when tested.

### Cognitive outcomes

To reduce the dimensionality of the cognitive data, composite age-corrected measures of fluid and crystallized intelligence were taken from the NIH Toolbox in the ABCD Neurocognition Battery^21^. Sum scores of fluid abilities were computed from the Dimensional Change Card Sort (DCCS) Test, the Flanker Inhibitory Control and Attention Test, the Picture Sequence Memory Test, the List Sorting Working Memory Test, and the Pattern Comparison Processing Speed Test. The fluid outcome therefore encompassed EF functions of inhibitory control (flanker), shifting (DCCS), and working memory (list sorting). Crystallized abilities were taken from the Picture Vocabulary Test and the Oral Reading Recognition Test.

### Correlational analysis

**Figure 1:**
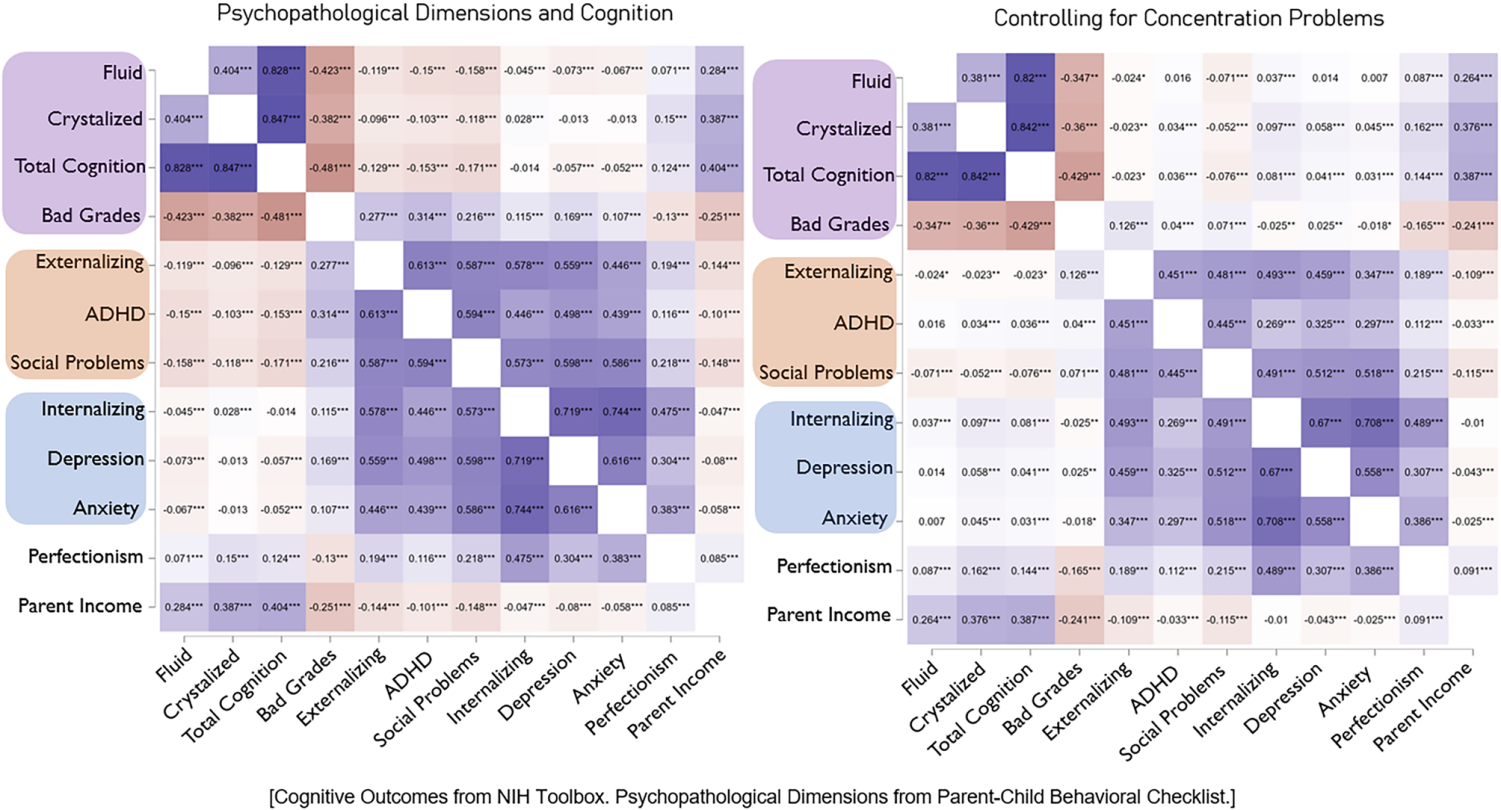
Associations between psychopathological dimensions and cognition. Pearson’s R Correlation Matrixes: Includes parent ratings of different psychopathological dimensions in children, cognitive performance, parent income, and educational outcomes. ***p < 0.001.

Pearson’s partial correlations were used to interrogate the interrelationships between cognitive outcomes and psychopathological dimensions (Fig. 1). A similar Pearson’s partial correlations analysis was used while also controlling for concentration difficulties (panel B). Bonferroni correction was used to control for multiple comparisons for both correlational analyses.

### Psychiatric stratifications and comparisons between high and low scorers in adhd and depression traits

For this analysis, psychiatric dimensions were stratified, taking the top 15% and bottom 15% percentiles of different dimensions to examine the hypothesis that dysregulated attention might be the primary factor underlying cognitive deficits. For depression and ADHD: (1) high depression and high ADHD, (2) high depression and low ADHD, (3) low depression and high ADHD, (4) low depression and low ADHD, and (5) other (i.e. controls). Similarly, for anxiety and ADHD: (1) high anxiety and high ADHD, (2) high anxiety and low ADHD, (3) low anxiety and high ADHD, (4) low anxiety and low ADHD, and (5) other (i.e. controls). Descriptive statistics for each stratification can be seen in the supplementary materials (S13).

**Figure 2:**
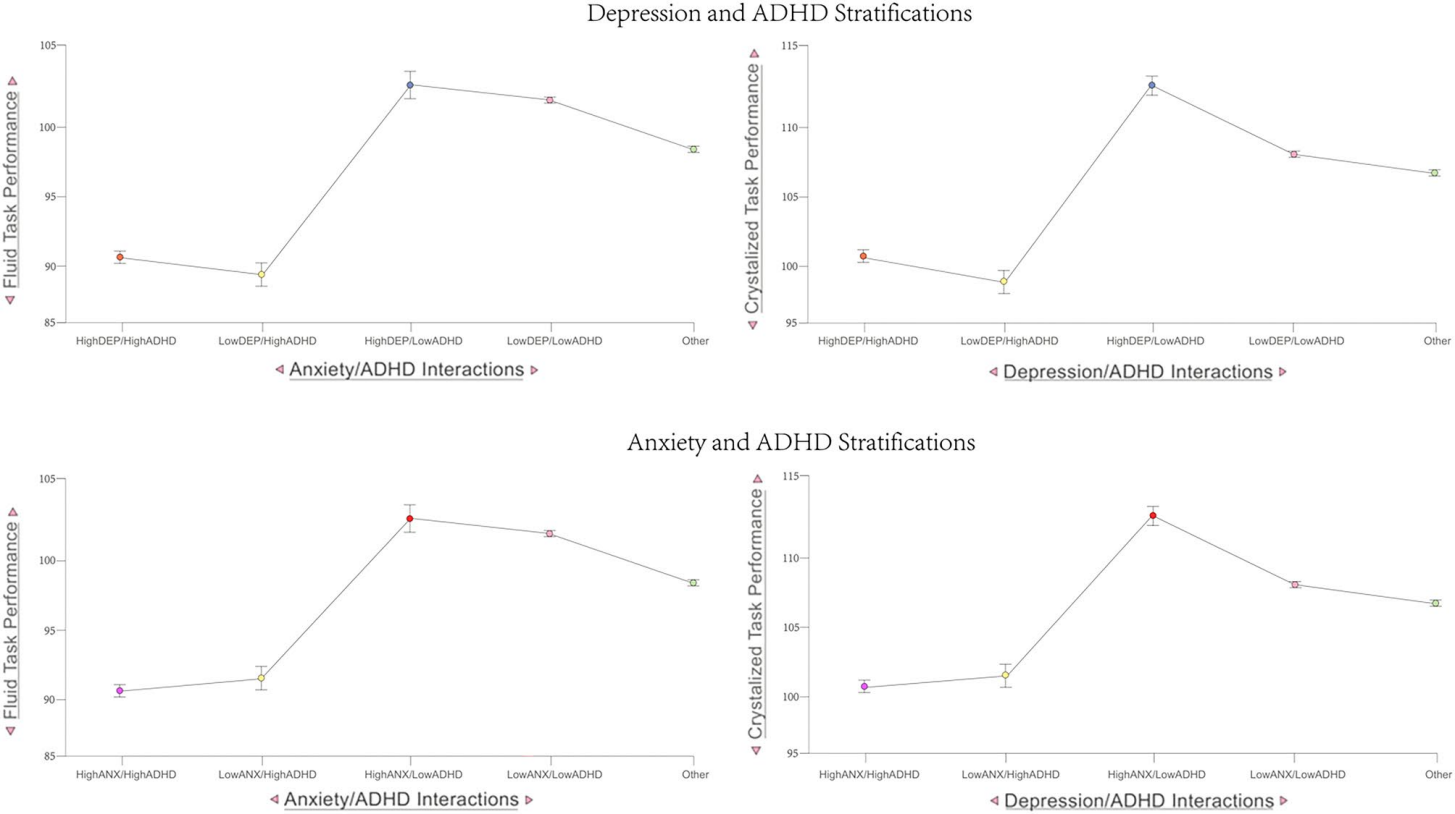
Stratifications (ADHD, depression, and anxiety dimensions) and fluid/crystalized cognition. Outcomes of fluid and crystalized cognitive performance across psychiatric stratifications of the top 15% and lower 15% percentiles of anxiety, depression, and ADHD dimensions. Individuals with high symptoms of depression or anxiety but low in the ADHD dimension exhibit comparable fluid task performance to controls and people low in both anxiety/depression and ADHD dimensions, and even out-perform these stratifications in crystallized intelligence measures. For list of cognitive tasks in Fluid and Crystalized outcome scores see the “Methods” section. Error bars denote SE.

One-way ANCOVAs were performed to compare the effect of these different psychopathological stratifications on the tasks measuring fluid, crystallized, and total cognitive performance separately for depression and anxiety stratifications (Fig. 2). The Supplementary materials include individual task outcomes and other associated psychopathological and environmental variables of these stratifications. Psychiatric stratifications and sex were treated as fixed factors, while parental income and stage of puberty were treated as covariates of no interest. The first group of two-way ANOVAs were then conducted to assess whether parental income, concentration difficulties, or an interaction of the two variables affects fluid, crystallized, and total cognitive performance. The second group of two-way ANOVAs were conducted to assess the impact of high depression and high anxiety psychiatric stratifications, concentration difficulties, or an interaction of these variables on fluid, crystallized, and total cognitive functioning. Since SES had a robust and linear relationship with cognitive outcomes, we also stratified individuals based on their magnitude of attention problems within household incomes to examine how attention dysregulation might show differential relationships with cognitive deficits across SES strata.

Tukey’s HSD post hoc tests were performed on any significant omnibus test for interrogating specific group differences and Bonferroni corrected for multiple comparisons. All models were tested for internal-consistency reliability via odd–even split-half analysis; these results are included in the Supplemental materials (S12).

### Gaussian graphical models of psychopathological dimensions

Gaussian graphical models (GGMs) were used for with edges parameterized as partial correlation coefficients controlling for every other variable in the network. In a confirmatory analysis, we analyzed the centrality of dimensions related to attention dysregulation, hypothesizing the central place of ADHD symptoms of impulsivity, poor concentration, hyperactivity and being easily distractible. In contrast, exploratory analysis was used to examine statistical relationships among different psychopathological features and indicate possible bridging traits between broader categories of ADHD, low mood, anxiety, oppositional defiant disorder (ODD), and negative peer relationships. Two GGMs were implemented to examine how well connections and centrality measures of psychopathological dimensions scaled with the size of the network. Further, two different classification schemes were used to classify clusters of variables (Fig. 4) given there is disagreement about their theoretical usefulness in nosology^22^. Figure 4A network is based on DSM-oriented classification schemes. In contrast, Fig. 4B network is based on the Hierarchical Taxonomy of Psychopathology (HiTop), which makes some attempts to better model and classify psychopathology symptoms given the substantial comorbidity, heterogeneity, unreliability, and arbitrary cutoffs in the DSM. Thresholds for the DSM and HiTOP networks were set to 0.1, wherein edges with an absolute value below that partial correlation coefficient were not shown in the network.

Centrality metrics are an important output of the GGM models (Fig. 4). Betweenness is the quantified outcome of how frequently a node lies on the shortest path connecting any two nodes^23^. Expected influence measures the sign of all edge weights, accounting for the presence of negative edges^15^.

We used a Case-dropping Bootstrap network (nBoots = 1000) to assess the stability of edge and centrality scores^24^. This method works by dropping subsets of the sample by 10% to see how well scores are retained. The supplementary materials section contains the outcomes and tables of the exact different centrality and clustering measures per variable.

### Directed-acyclic graph of psychopathological dimensions

Finally, a directed-acyclic graph (DAG) employing a Bayesian hill-climbing method was used to supplement the partial correlation network to indicate any possible directionality, common causes, and colliders among psychopathological dimensions (Fig. 5). Following the recent recommendations on longitudinal network analysis^25,26^, we ran Ordinary Least Squares (OLS) regression to estimate the (linear) slope of the node scores across four observation time points using complete cases. The value of the slope represents each participant’s change over time on a given item. We then used the derived slope values to estimate the causal structure using a DAG. DAGs provide information not only about the strength but also about the direction of connections between nodes^27^. As the DAG is directed and has no feedback loop (i.e. acyclic), it provides preliminary inference about the causal relationships among nodes^28^. In DAG, upstream nodes have predictive priority and may be considered the causes of downstream nodes^29^. Specifically, the DAG was modelled via the Hill-Climbing algorithm using the hc() function of the *bnlearn* package^30^. We took three steps to ensure model stability^27^. First, we ran 50 different random starts to avoid local maxima and 100 perturbations to iteratively insert, remove, or reverse an edge to determine the best-fitting structure of the DAG-based on the optimization of the goodness-of-fit index (i.e. BIC values). Doing so allows us to generate an initial DAG with our data. Second, we bootstrapped 500 DAGs to obtain edge frequency and compute the significance, direction, and strength of the edges^16^. Specifically, if an edge is above an empirical threshold estimated using the bootstrapped networks, it is considered statistically significant and therefore will be retained^27^. This approach ensures a good balance between sensitivity and specificity. The final direction of the retained edges was then determined using majority voting, that is, the direction of the arrow that appears in at least 70% of the 500 bootstrapped networks (See supplementary materials for 51%). The thickness of each edge indicates the percentage of the times the edge goes in the direction depicted in the final visualization.

## Results

The interrelationships between cognition and psychopathological symptom dimensions are shown in Fig. 1. There were statistically significant correlations between cognitive performance and ADHD dimensions, depressive and anxious dimensions, internalizing and externalizing and perfectionism, with correlation co-efficients ranging in size between 0.01 and 0.15 (between ADHD traits and total cognition).

### ANCOVA analyses on psychopathological stratifications

A one-way ANCOVA with parental income and stage of puberty as covariates revealed statistically significant differences on all three measures of cognitive performance between ADHD and depression stratifications (Tables 1,2); those with high depression and high ADHD, and those with low depression and high ADHD stratifications showed significant impairment (Fig. 2). Similar patterns are seen among task scores for individual EF tasks within the fluid task performance measure across the ABCD neurocognition battery (Supplementary Materials S1, S2, S3). The results for all *post-hoc* tests for ADHD/Depression stratifications can be viewed in S1. Odd–even split-half analyses of the full sample confirmed these results were internally consistent (Supplemental material Table 13).

**Table 1:** One-way ANCOVA results of ADHD/Depression stratifications on types of cognitive performance (top = fluid; middle = crystallized; bottom = total).

Cognitive performance type	Df	F	P-value
Fluid	4, 8501	26.092	< 0.001***
Crystallized	4, 15,065	17.069	< 0.001***
Total	4, 8506	26.820	< 0.001***

*Df* degrees of freedom, *F* F-statistic.

P-value  significance level of test. *p < 0.05, **p < 0.01, ***p < 0.001.

**Table 2:** Tukey’s post-hoc tests assessing group differences in depression/ADHD psychopathological stratifications.

		Mean difference	95% CI for mean difference		SE	t	p_tukey_	p_bonf_
			Lower	Upper				
HighDEP, HighADHD	LowDEP, HighADHD	1.484	− 2.342	5.311	1.403	1.058	0.828	1.000
	HighDEP, LowADHD	− 5.804	− 9.137	− 2.471	1.221	− 4.752	< 0.001***	< 0.001***
	LowDEP, LowADHD	− 4.821	− 6.879	− 2.762	0.755	− 6.389	< 0.001***	< 0.001***
	Other	− 2.144	− 4.165	− 0.124	0.740	− 2.896	0.031*	0.038*
LowDEP, HighADHD	HighDEP, LowADHD	− 7.288	− 11.603	− 2.973	1.582	− 4.608	< 0.001***	< 0.001***
	LowDEP, LowADHD	− 6.305	− 9.733	− 2.877	1.256	− 5.018	< 0.001***	< 0.001***
	Other	− 3.629	− 7.035	− 0.222	1.249	− 2.906	0.030*	0.037*
HighDEP, LowADHD	LowDEP, LowADHD	0.983	− 1.871	3.837	1.046	0.940	0.881	1.000
	Other	3.660	0.829	6.490	1.038	3.527	0.004**	0.004**
LowDEP, LowADHD	Other	2.676	1.604	3.749	0.393	6.806	< 0.001***	< 0.001***
HighDEP, HighADHD	LowDEP, HighADHD	3.006	0.250	5.762	1.010	2.975	0.024*	0.029*
	HighDEP, LowADHD	− 5.489	− 7.946	− 3.032	0.901	− 6.094	< 0.001***	< 0.001***
	LowDEP, LowADHD	− 1.176	− 2.693	0.341	0.556	− 2.115	0.214	0.345
	Other	− 0.398	− 1.877	1.081	0.542	− 0.734	0.949	1.000
LowDEP, HighADHD	HighDEP, LowADHD	− 8.495	− 11.625	− 5.365	1.147	− 7.404	< 0.001***	< 0.001***
	LowDEP, LowADHD	− 4.182	− 6.644	− 1.720	0.902	− 4.633	< 0.001***	< 0.001***
	Other	− 3.404	− 5.843	− 0.964	0.894	− 3.807	0.001**	0.001**
HighDEP, LowADHD	LowDEP, LowADHD	4.313	2.198	6.428	0.775	5.563	< 0.001***	< 0.001***
	Other	5.091	3.000	7.182	0.766	6.643	< 0.001***	< 0.001***
LowDEP, LowADHD	Other	0.778	− 0.020	1.576	0.292	2.660	0.060	0.078
HighDEP, HighADHD	LowDEP, HighADHD	0.185	− 0.025	0.395	0.077	2.404	0.114	0.162
	HighDEP, LowADHD	− 0.389	− 0.572	− 0.206	0.067	− 5.807	< 0.001***	< 0.001***
	LowDEP, LowADHD	− 0.204	− 0.316	− 0.091	0.041	− 4.919	< 0.001***	< 0.001***
	Other	− 0.094	− 0.205	0.017	0.041	− 2.319	0.139	0.204
LowDEP, HighADHD	HighDEP, LowADHD	− 0.574	− 0.811	− 0.337	0.087	− 6.617	< 0.001***	< 0.001***
	LowDEP, LowADHD	− 0.388	− 0.577	− 0.200	0.069	− 5.638	< 0.001***	< 0.001***
	Other	− 0.279	− 0.466	− 0.092	0.068	− 4.076	< 0.001***	< 0.001***
HighDEP, LowADHD	LowDEP, LowADHD	0.185	0.029	0.342	0.057	3.233	0.011*	0.012*
	Other	0.295	0.140	0.450	0.057	5.181	< 0.001***	< 0.001***
LowDEP, LowADHD	Other	0.109	0.051	0.168	0.022	5.072	< 0.001***	< 0.001***

Top: fluid, middle: crystallized, bottom: total.

Mean difference the mean difference between groups, Lower lower bound 95% confidence interval, Upper upper bound 95% confidence interval, *SE* standard error, Ptukey p-value of given post-hoc test (uncorrected for multiple comparisons), Pbonf p-value of given post-hoc test (corrected for multiple comparisons). *p < 0.05, **p < 0.01, ***p < 0.001.

Likewise, a one-way ANCOVA with the same covariates showed statistically significant differences in all cognitive performance types between ADHD/Anxiety stratifications (Table 3). In a similar pattern to our ADHD/depression stratifications, those in the high anxiety/high ADHD and those in the low anxiety/high ADHD stratifications showed significant impairment on all three measures of cognitive performance (Fig. 2). The results for all post-hoc tests for ADHD/Anxiety stratifications can be viewed in Table 4. Odd–even split-half analyses of the full sample confirmed these results were internally consistent (S13).

**Table 3:** One-way ANCOVA results of ADHD/anxiety stratifications on types of cognitive performance (top = fluid; middle = crystallized; bottom = total).

Cognitive performance type	Df	F	P-value
Fluid	4, 8501	20.423	< 0.001***
Crystallized	4, 15,065	15.757	< 0.001***
Total	4, 8500	17.483	< 0.001***

*Df* degrees of freedom; *F* F-statistic.

P-value significance level of test. *p < 0.05, **p < 0.01, ***p < 0.001.

**Table 4:** Tukey’s post-hoc tests assessing group differences in anxiety/ADHD psychopathological stratifications.

		Mean difference	95% CI for mean difference		SE	t	p_tukey_	p_bonf_
			Lower	Upper				
HighANX, HighADHD	LowANX, HighADHD	2.284	– 1.699	6.267	1.460	1.565	0.520	1.000
	HighANX, LowADHD	– 5.311	– 8.470	– 2.152	1.158	– 4.587	<0.001***	< 0.001***
	LowANX, LowADHD	– 4.741	– 6.936	– 2.545	0.805	– 5.892	<0.001***	< 0.001***
	Other	– 2.101	– 4.213	0.012	0.774	– 2.713	0.052	0.067
LowANX, HighADHD	HighANX, LowADHD	– 7.595	– 11.809	– 3.381	1.545	– 4.917	<0.001***	< 0.001***
	LowANX, LowADHD	– 7.025	– 10.578	– 3.471	1.302	– 5.394	<0.001***	< 0.001***
	Other	– 4.385	– 7.891	– 0.879	1.285	– 3.412	0.006**	0.006**
HighANX, LowADHD	LowANX, LowADHD	0.570	– 2.019	3.160	0.949	0.601	0.975	1.000
	Other	3.210	0.684	5.736	0.926	3.467	0.005**	0.005**
LowANX, LowADHD	Other	2.640	1.523	3.757	0.409	6.451	<0.001***	< 0.001***
HighANX, HighADHD	LowANX, HighADHD	1.958	– 0.984	4.899	1.078	1.816	0.364	0.694
	HighANX, LowADHD	– 5.506	– 7.852	– 3.160	0.860	– 6.403	< 0.001***	< 0.001***
	LowANX, LowADHD	– 1.280	– 2.897	0.336	0.593	– 2.160	0.195	0.308
	Other	– 0.701	– 2.254	0.852	0.569	– 1.231	0.733	1.000
LowANX, HighADHD	HighANX, LowADHD	– 7.464	– 10.594	– 4.334	1.147	– 6.506	< 0.001***	< 0.001***
	LowANX, LowADHD	– 3.238	– 5.868	– 0.608	0.964	– 3.359	0.007**	0.008**
	Other	– 2.659	– 5.251	– 0.066	0.950	– 2.798	0.041*	0.052
HighANX, LowADHD	LowANX, LowADHD	4.226	2.290	6.161	0.709	5.957	< 0.001***	< 0.001***
	Other	4.805	2.920	6.691	0.691	6.953	<0.001***	< 0.001***
LowANX, LowADHD	Other	0.580	– 0.245	1.404	0.302	1.918	0.307	0.551
HighANX, HighADHD	LowANX, HighADHD	0.129	– 0.089	0.347	0.080	1.612	0.490	1.000
	HighANX, LowADHD	– 0.359	– 0.533	– 0.186	0.063	– 5.659	< 0.001***	< 0.001***
	LowANX, LowADHD	– 0.208	– 0.328	– 0.087	0.044	– 4.708	< 0.001***	< 0.001***
	Other	– 0.099	– 0.215	0.017	0.042	– 2.339	0.133	0.193
LowANX, HighADHD	HighANX, LowADHD	– 0.488	– 0.719	– 0.257	0.085	– 5.765	<0.001***	< 0.001***
	LowANX, LowADHD	– 0.337	– 0.532	– 0.142	0.071	– 4.714	< 0.001***	< 0.001***
	Other	– 0.228	– 0.421	– 0.036	0.070	– 3.241	0.011*	0.012*
HighANX, LowADHD	LowANX, LowADHD	0.152	0.010	0.294	0.052	2.913	0.029*	0.036*
	Other	0.260	0.121	0.399	0.051	5.120	< 0.001***	< 0.001***
LowANX, LowADHD	Other	0.108	0.047	0.170	0.022	4.828	< 0.001***	< 0.001***

Top: fluid, middle: crystallized, bottom: total.

Mean difference the mean difference between groups, Lower lower bound 95% confidence interval, Upper upper bound 95% confidence interval, *SE* standard error, Ptukey p-value of given *post-hoc* test (uncorrected for multiple comparisons), Pbonf p-value of given *post-hoc* test (corrected for multiple comparisons). *p < 0.05, **p < 0.01, ***p < 0.001.

### Effects of parental income, depression and anxiety, and concentration difficulties on cognitive performance

**Figure 3:**
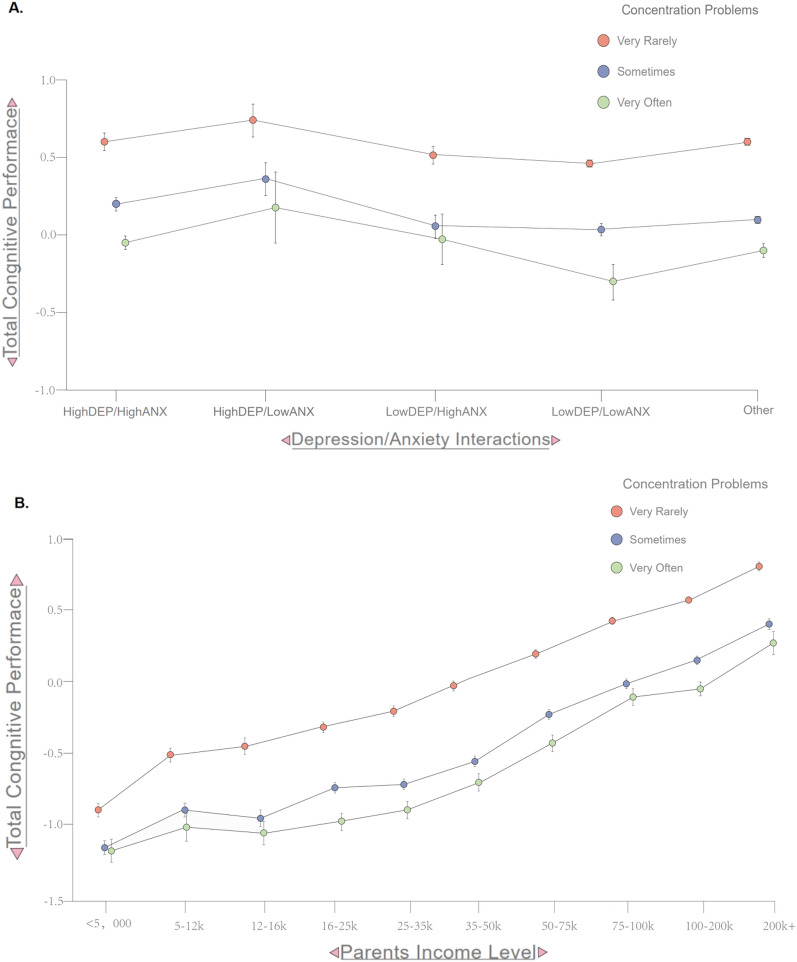
Attention dysregulation accounts for significant variation in cognitive performance among different anxiety/depression phenotypes and different SES levels. Panel (**A**) shows total cognitive performance (as aggregation of fluid and crystalized measures) across different psychiatric stratifications of anxiety and depression with different lines for concentration problems. Panel (**B**) shows total cognitive performance as a function of parental income with different lines for degree of concentration problems. Error bars denote SEM.

Three 2-way ANOVAs showed significant main effects of parental income, depressive and anxious symptomatology, and concentration difficulties on our measures of cognitive performance; with parental income and concentration difficulties significantly influencing all three types, while depression and anxiety influencing only crystallized and total cognitive performance (Table 5; see also Fig. 3). No significant interaction effects were identified between parental income, depression and anxiety, or concentration difficulties on any measure of cognitive performance (p > 0.05). Odd–even split-half analyses of the full sample confirmed these results were internally consistent (Supplemental material Table 13).

**Table 5:** 2-way ANOVA results of parental income (top), depression and anxiety symptomatology (middle), and concentration difficulties (bottom) on types of cognitive performance (top = fluid; middle = crystallized; bottom = total).

Predictor	Cognitive performance type	Df	F	P-Value
Parental income	Fluid	9, 10,503	46.947	< 0.001***
	Crystallized	9, 17,407	165.778	< 0.001***
	Total	9, 10,562	113.763	< 0.001***
Depression & anxiety	Fluid	N/A	N/A	> 0.05
	Crystallized	9, 17,407	165.778	< 0.001***
	Total	9, 11,125	3.796	0.004**
Concentration difficulties	Fluid	9, 10,503	46.947	< 0.001***
	Crystallized	9, 17,407	165.778	< 0.001***
	Total	9, 10,562	113.763	< 0.001***

*Df* degrees of freedom; *F* F-statistic.

P-value significance level of test. *p < 0.05, **p < 0.01, ***p < 0.001.

### Partial correlation network outcomes

**Figure 4:**
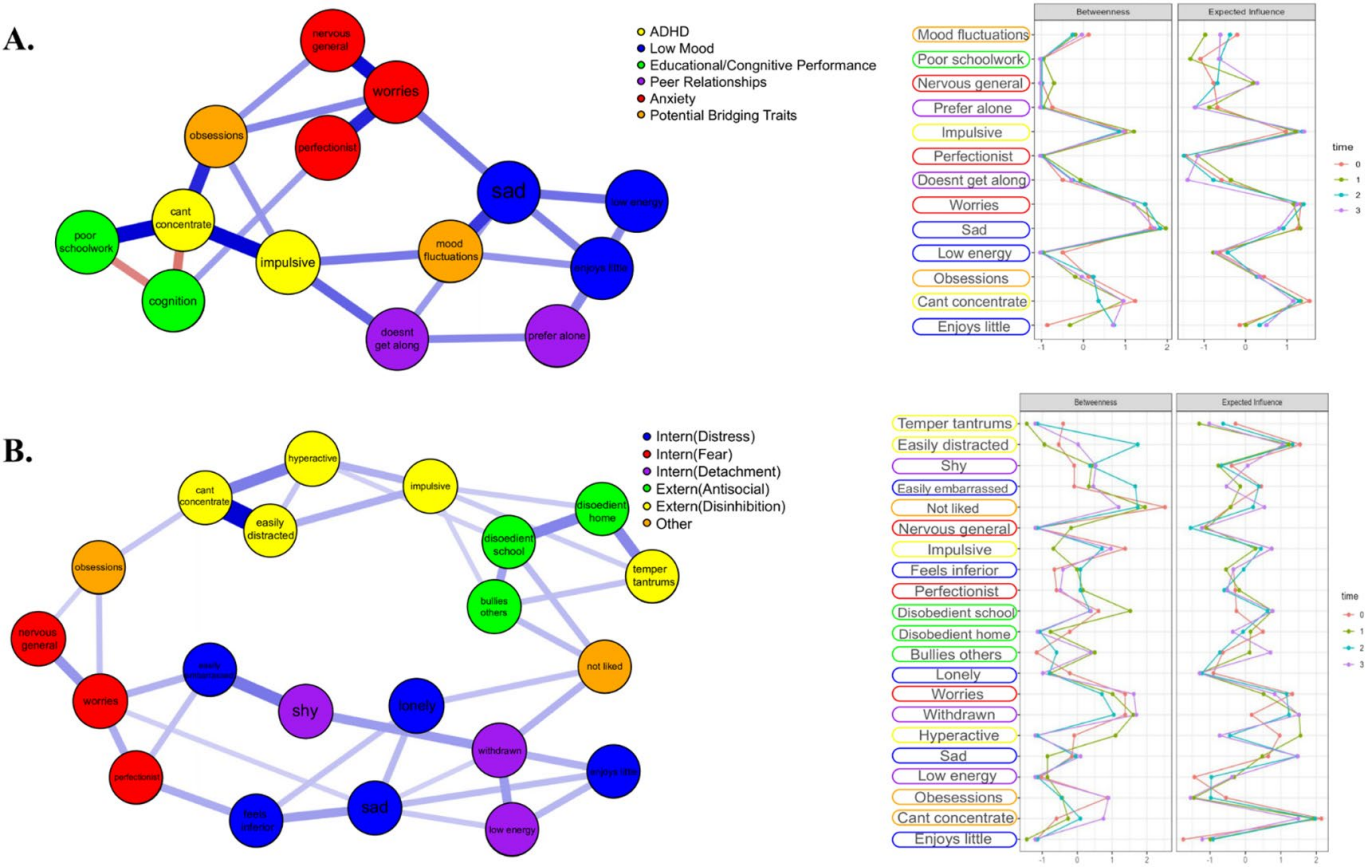
Dynamic interaction of symptoms and centrality of attention dysregulation. Partial Correlation Networks for psychopathological variables using parent ratings from the CBCL. Panel (**A**) shows a smaller DSM-oriented classification scheme with hallmark ADHD, depression, and anxiety symptoms. Panel (**B**) shows a larger dimension of symptoms and is classified based on the HiTop classification scheme. Centrality measures on the right side of the diagram show the computed centrality metrics for each psychopathological dimensions across each of the time points at which data were collected.

ADHD associated dimensions had the highest expected influence scores (sign of all edge weights with other nodes), alongside hallmark symptoms of anxiety (worry) and low mood (sadness) in the smaller network (Fig. 4A). Exploratory findings indicated obsessive thinking styles as a potential bridge between ADHD dimensions and anxiety symptoms (Fig. 4A,B) whereas negative peer relationships were more prominent as a bridge between ADHD/ODD and social detachment/distress (Fig. 4B), which conceivably facilitated low mood.

### Node selection and display

Although centrality measures show variables at different time points, GGM edges were aggregated for simplicity across the four time points collected. Network connections remained considerably stable across the four time points at the current threshold, however the sparsity of edges showed a slight decrease over time. The total cognitive performance node was left out of the centrality measures because it was collected at fewer time points and had low centrality because psychopathological traits were considerably more connected to each other than to cognition.

### Hierarchical influence of attention dysregulation

**Figure 5:**
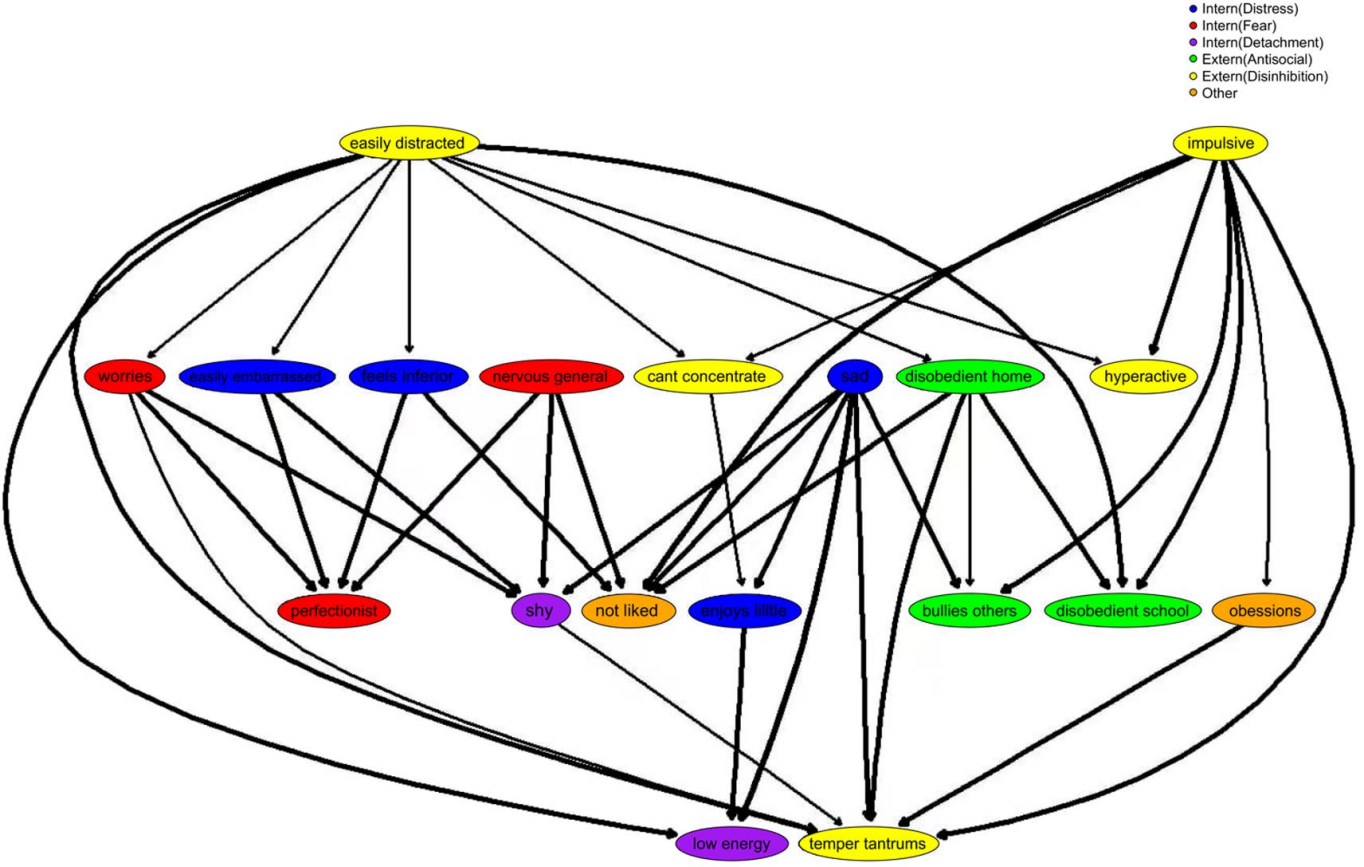
Bayesian network. A Bayesian network (directed acyclic graph; DAG) depicting symptoms of ADHD, Low Mood, Anxiety, ODD, and peer relationships using hill-climbing method^27^. See the “Methods” section for the specific protocol and supplement (S11) for less conservative threshold.

To supplement the GGM and indicate potential causality among psychopathological features we ran the same variables from network B in a Directed Acyclic Graph, seen in Fig. 5, employing a Bayseian Hill-Climbing Method to determine the graph structure. Certain ADHD traits – easily distracted, impulsive – are seen at the top of the Directed Acyclic Graph of Psychopathological Structure Employing Bayesian Hill-Climbing Method (Fig. 5), indicating the potential for predictive priority and possible causal influence on both internalizing and externalizing traits. Being nervous in general and sadness also appeared influential nodes, with either direct or indirect effects on internalizing and externalizing traits.

## Discussion

Our two main hypotheses, that (1) attentional dysregulation would underpin transdiagnostic general cognitive impairments, and (2) be strongly associated (and perhaps causative) with other wide-ranging psychopathological features, were supported by the findings of this study. Discussion of each hypothesis is subsequently presented sequentially.

### Attention dysregulation as a central component in transdiagnostic cognitive deficits

Consistent with our first hypothesis, these results suggest that previous findings of transdiagnostic cognitive impairments may largely be a function of an individual’s degree of attentional dysregulation, especially within dimensions of anxiety or low mood (see Figs. 1,2). Although evidence of general transdiagnostic cognitive dysfunction in psychopathology was consistently significant in this large cohort, it was small^31^ and was not observable in unidimensional traits of low mood or anxiety^32^, especially when controlling for the inattention component of ADHD (Fig. 1). In fact, individuals experiencing symptoms of anxiety or depression without the ADHD dimension not only exhibited typical fluid and closely related EF cognitive performance (S1), but outperformed controls in tasks assessing crystalized intelligence (Fig. 5, Table 5). Therefore, consistent with one of our primary objectives, converging associative, stratification, and network analytic findings all indicated that components of attentional dysregulation may be a central component of poor performance in cognitive tasks (see Figs. 1, 2, 3, 4A, S10). These findings have important implications for future research examining the relationship of cognition and psychopathology. Namely, if attempts are made to measure cognitive deficits in psychopathological dimensions or clinical populations, researchers should closely screen for co-occurring attention problems.

Unfortunately, regulatory systems controlling attention and transient affective perturbations are tightly inter-related so that it is often difficult to delineate whether cognitive impairments represent a potential co-morbidity or state-specific effects of negative affect. Nevertheless, externalization traits accounted for most of the diminished educational and cognitive outcomes, which were largely accounted for by the dimension of attention dysregulation (Fig. 1). Parental income (as a proxy for SES) had a large and markedly linear effect on cognitive outcomes (see Fig. 3B). Even so, individual attentional capacities accounted for a large degree of variation in this relationship. Attention dysregulation could transiently and synergistically be exacerbated by low mood or anxiety but they may not be indicative of dysfunctional cognition within certain ranges. Poorly regulated attentional systems in those with a predisposition to low mood or anxiety could impair recovery speed^33^, and may generally exacerbate the susceptibility to aversive state changes due to the maintenance and quality of internal coping strategies implemented, as well as the effect they have on ongoing negative environmental interactions. However, some cognitive biases characteristic of unidimensional anxiety or low mood may be predominantly self-referential in their processing.

The (exploratory) finding here that trait perfectionism might buffer against some of the cognitive and educational impairments of other psychopathological traits (Fig. 4, S4) may represent individual’s higher regard for others’ evaluations, and hence more intrinsic motivation towards better task performance. Moderate levels of trait worry actually improved cognitive performance in a classic inverted *U* shape (S3), and high levels of trait perfectionism enhanced the positive relationship between anxiety with cognitive performance and educational outcomes (S4).

Therefore, hallmark traits of low mood (sadness) and anxiety (worry) may not impair cognitive or educational performance unless they are severe to the point of exhausting cognitive/motivational resources or co-existing with the attentional dysregulation that is paradigmatic of ADHD. Extreme depressive episodes may causally disrupt otherwise normal cognitive functions through self-reinforcing feedback in complex biopsychological systems^34^. However, even when depressive symptoms met diagnostic thresholds as rated by external clinicians, concentration problems still accounted for substantial variation in cognitive and educational outcomes (S5, S6).

There are two prospective explanations for the moderating influence of attention dysregulation on cognitive performance seen here. One is that individual self-motivational systems supporting the maintenance of normative task demands and goal-directed action may be hypofunctional^35,36^, especially in tasks deemed tedious. Signs of attention dysregulation could indicate high levels of reinforcement may be needed to motivate performance. In some cases, this could represent a disconnect between knowledge and performance^7^. Individuals with ADHD commonly exhibit hyper-focus on tasks for which they have high intrinsic idiographic motivation^37^. Robust connections between cognitive control and motivation have been extensively documented^38^, making this a straightforward link to potential impairments in cognitive tasks. Indeed, the dimensional proxy for anhedonia or motivational deficits (“enjoys little”) had among the highest association with task performance within the DSM-oriented depression symptom checklist.

A more classical, and not entirely incompatible possible explanation is that attention problems may be a significant proxy for more generally dysregulated cognitive control systems. Features of ADHD may have some adaptive value in specific environments, and whereas cognitive control is a balance between flexibility and maintenance of information^39,40^, extremes of the latter can also be maladaptive and generate ‘attention surplus’^12^. However, developmental delays in cortical maturation seen in ADHD^41–43^ might support this explanation. Attention has been described as a design feature for efficient goal-directed action^6^ and is a central neural control feature subserving agential efficacy. Consequently, the impairments characteristic of attention dysregulation could involve dysfunctional input-gating mechanisms for filtering contextually relevant information, failures of dopamine to maintain task representations in working memory^44^, or inefficient output-gating mechanisms precipitating the impulsivity component, frequently manifested as sub-optimal premature responding.

### Network modelling of the centrality, connections, directionality, and possible hierarchical influence of attention dysregulation

Our other primary aim was to identify the centrality and potential hierarchical influence of attention dysregulation in broader psychopathological dimensions, particularly for anxiety and low mood. Partial correlation and Bayesian networks both synergistically supported the possibility of attention dysregulation having strong diverse associations with, and perhaps causally affecting disparate psychopathological dimensions (see Figs. 4, 5). Further, corroborating prior reserach, stratification analysis indicated that ADHD features, when coupled with anxiety or depression symptoms, strongly exacerbate other wide-ranging negative dimensions (S7), and have a high expected influence in broader psychopathological networks^45,46^.

Regarding the analysis depicted in Figs. 4, 5, there are two important novel implications of the different network analytic approaches implemented, one of which was confirmatory and the other exploratory. Confirmatory analysis indicated the centrality and influence of attention dysregulation (concentration problems, distractibility, and increasingly so with impulsivity across time points) scaled with the size and different theoretical assumptions of the networks, signifying its likely impact on a wide range of other psychopathological features. Therefore, the stability of the expected influence of attention dysregulation proxies in the networks across time points, scales, and sample sizes suggests its importance in the maintenance and stability of different psychopathological dynamics (Fig. 4, S9, S10, S11).

In exploratory analysis, examining node relationships (Fig. 4), two potentially important bridging trajectories were indicated in the networks between ADHD dimensions and hallmark symptoms of depression & anxiety. One of these paths involved the inattention component of ADHD linking to obsessive thinking, which bridged to paradigmatic features of anxiety, as well as perfectionism and feelings of inferiority. The other consisted of traits more strongly connected to the impulsivity component of ADHD and traits related to disinhibited externalizing and general disobedience/defiance, which appeared to exacerbate negative peer relationships, and may straightforwardly be a bridge to feelings of loneliness and low mood more broadly.

Unsurprisingly, anxiety and low mood symptoms were strongly interconnected. Desensitization of affective systems through increased exposure to aversive events could increase co-morbidity between aversive states of anxiety and depression^47^. Further, it is potentially the case that anxiety develops into low or severe mood once there is a realization that salient goals can no longer be potentially achieved, which is probably common in individuals with baseline dysregulated attentional systems.

Many of these dimensions likely exist in feedback loops and it is difficult to infer a potential direction of causation or distinguish causes from effects in an undirected network. Therefore, whether attentional dysregulation is a cause, effect, or exerts a bidirectional influence on psychopathological dynamics is unclear from GGMs alone. It is undoubtedly the case that stressful environmental factors and internal symptoms of sadness, anhedonia, and obsessive thinking could impair the capacity for sustained attention in normative domains. Rumination processes may in fact increase sustained attention, but only within narrow domains of internal distress relating to idiosyncratic goals, which dimensions of ADHD could potentially amplify. However, the maintenance and execution of contextually appropriate goals despite environmental adversity or internal distress may actually otherwise be facilitated by moderate anxiety or trait perfectionism.

The results of Bayesian hill-climbing analysis applied to a psychopathology network resulted in a Directed Acyclic Graph of psychopathology (Fig. 5). Upstream symptoms in DAGs are likely candidates for interventions^16^. The analysis indicated that attentional dysregulation, impulsivity, and sadness may potentially be causally related to a range of aversive outcomes, further suggesting they are important targets for intervention. As previously found, ADHD traits and sadness (likely a proxy for distress) strongly influence a range of other psychopathological outcomes^16^. Here again, in a much larger sample, both network analytic approaches differentially converge on the importance of ADHD traits and sadness as important candidate psychopathological features that likely influence and structurally maintain a range of psychopathological symptoms.

Such interactions remain complex, but symptoms of ADHD do often precede many other disorders^8^, including those with which it shares a significant degree of co-morbidity, such as mood and anxiety disorders^17^, further suggesting a potential directionality of causal influence in some cases^10^. Impulsivity and attentional dysregulation components of ADHD may potentially represent more ‘essential’ neurobiological mechanisms, developing within a relatively stable temporal trajectory^48^. In contrast, sadness or worry likely depends more on situationally dependent intentional content, involving more variable temporal fluctuations.

Of critical clinical importance, the concatenation of ADHD and internalization symptoms of distress greatly increase rates of highly adverse outcomes^45^. A subsequent exploratory analysis here corroborated this, showing co-occurring symptoms of ADHD and low mood (compared to either dimension alone) in individuals were associated with a range of other adverse and severe psychopathological features (especially aggressive and attention-seeking tendencies), negative peer relationships, several parental pathologies and drug use, and several outcomes on the parent-rated Barkley’s Executive Functioning scale. (See supplementary material S7, S8). Therefore, consistent with our results and others, impulsivity and/or attention dysregulation in adolescents could be central in the nexus of wide-ranging normative cognitive or social impairments & relationships, which could interactively feedback with low mood or anxiety – making it a cardinal clinical target for early interventions^49,50^.

### Limitations

Several important limitations should be noted. Firstly, the most noteworthy limitation is that the primary metrics of psychopathology were *parental* ratings of their children. Compared to clinicians, parents do have much more exposure to their children and hence can assess their attentional capacities in many contexts. Nevertheless, such ratings could have been influenced by any number of parent–child measurement biases^51^. However, these measures of concentration were in fact strongly associated with educational performance and did still account for much variance among objective measures of cognition which supports their validity. Metrics of attention dysregulation still appeared to account for large variation across SES strata. Precise mechanisms are of course difficult to infer from self-report measures, but we believe that the many converging analyses provide highly suggestive support of our primary hypothesis and appear to have potentially important implications.

Subjective measures and cognitive tasks which are purported to measure the same psychological construct often don’t correlate well^52,53^. While some potential explanations were provided above, the conceptual relationship between parental reports of attentional dysregulation and observable cognitive deficits is difficult to precisely ascertain. One possibility is that they map onto similar neurocognitive processes. Controlling for even more extensive wide-ranging psychopathological dimensions, concentration problems still have the closest relationship to fluid outcomes (S10). Still, attention dysregulation may be either a neurodevelopmental feature (which results suggest may strongly exacerbate other dimensions) or a consequence of severe negative affect. However, consistent with these analyses and others, while attention dysregulation or cognitive dysfunction may be an important risk factor, they are very likely not a necessary feature of dimensions of low mood or anxiety within some ranges, reflecting variable etiological pathways also seen elsewhere^54^.

Another significant limitation is that the age of onset of other categorically distinct anxiety and mood disorders typically come at later ages^8^, even if features of ADHD examined here are largely predictive of later mental health problems in those domains. Whereas anxiety can plausibly be conceived as a reasonably stable trait across the lifetime^55^, with some critical contextual fluctuations, there is often a tipping point in depressive episodes where symptoms can become severely debilitating, in part through self-reinforcing mechanisms. However, some adolescents in this data set did have a formal DSM diagnosis of MDD (N = 210), and concentration problems were still a substantial moderating factor in cognitive and educational outcomes among these individuals (S6, S7). Cognitive tasks examined here primarily relate to metrics of crystallized & fluid intelligence, with the latter subsuming EF (see S1 for specific EF task analyses). EF components have consistently shown negative associations with clinical ADHD, especially in children, but may not be the most ideal tasks to investigate cognitive impairments or biases in anxiety or depression. Nevertheless, anxiety and mood disorders have previously still been associated with deficits in these domains, though mostly in adults^1^.

## Conclusion

While general psychopathology might typically be associated with, and predictive of, cognitive impairments^1,56^, these results suggest that underperformance in a wide range of cognitive tasks in adolescents may be largely due to attentional dysregulation, especially in individuals experiencing anxiety or low mood (and perhaps depression in some cases). While the relationship between executive functions and general psychopathology was relatively small in this dataset, EF was still found to be a prospective predictor of change in the general psychopathology “P-factor” over time^57^, suggesting that EF impairments may take time to manifest into psychopathology. However, this current analysis indicates that specific phenotypes of individuals high in depressive or anxiety symptoms but low in ADHD remarkably outperform other groups in measures of crystalized task performance and are comparable in fluid/EF task performance and educational outcomes. Further, perfectionism and moderate worry were even associated with enhanced cognitive performance. These findings indicate that heterogeneous phenotypes and co-morbidities should be considered when making inferences about cognitive impairments in psychopathology. While symptoms of anxiety, low mood, and ADHD often exacerbate each other and relate in etiologically complex ways, the confirmatory analysis here indicated that attention dysregulation is an important component structurally maintaining and exacerbating other psychopathological features. Subsequent exploratory analysis indicated ADHD traits might be a common link and revealed potential bridging features that may lead to anxiety (via obsessive thinking) or low mood (via impulsivity, or negative peer relationships). Since these findings indicate a high centrality and potential direction of causation, early interventions targeting attention dysregulation may be helpful to diminish both the negative cognitive/social outcomes associated with ADHD and the synergistically worse pathological dynamics that occur when internalized distress is amplified.

## Supplementary Information

Supplementary Information.

## Data Availability

Data used in the preparation of this article were obtained from the Adolescent Brain Cognitive DevelopmentSM (ABCD) Study (https://abcdstudy.org), held in the NIMH Data Archive (NDA). The data used in this study are available from the NIMH Data Archive. This is a multisite, longitudinal study designed to recruit more than 10,000 children age 9-10 and follow them over 10 years into early adulthood. The ABCD Study® is supported by the National Institutes of Health and additional federal partners under award numbers U01DA041048, U01DA050989, U01DA051016, U01DA041022, U01DA051018, U01DA051037, U01DA050987, U01DA041174, U01DA041106, U01DA041117, U01DA041028, U01DA041134, U01DA050988, U01DA051039, U01DA041156, U01DA041025, U01DA041120, U01DA051038, U01DA041148, U01DA041093, U01DA041089, U24DA041123, U24DA041147. A full list of supporters is available at https://abcdstudy.org/federal-partners.html. A listing of participating sites and a complete listing of the study investigators can be found at https://abcdstudy.org/consortium_members/. ABCD consortium investigators designed and implemented the study and/or provided data but did not necessarily participate in the analysis or writing of this report. This manuscript reflects the views of the authors and may not reflect the opinions or views of the NIH or ABCD consortium investigators ABCD DOI: 10.15154/1527909.
