# Supplementary material for: Impact and centrality of attention dysregulation on cognition, anxiety, and low mood in adolescents
Source: Sci Rep. 2023 Jun 5;13:9106. doi: 10.1038/s41598-023-34399-y (PMC10241800; doi:10.1038/s41598-023-34399-y)
Supplement: Supplementary file 1 — Supplementary Information. [file 41598_2023_34399_MOESM1_ESM.docx]

# Supplementary Materials

## S1: Psychiatric Stratifications for Individual Executive Function Tasks

A one-way ANCOVA with parental income and stage of puberty as covariates revealed statistically significant differences of all three of the tasks specifically related to executive functions among ADHD/Depression stratifications (see Table and Figures below)

| Cognitive performance task | Df | F | P-Value |
| --- | --- | --- | --- |
| Flanker | 4, 15617 | 13.009 | <.001*** |
| Card Sorting | 4, 8682 | 6.938 | <.001*** |
| List Sorting | 4, 8666 | 12.241 | <.001*** |

Supplementary Table S1. One-way ANCOVA results of ADHD/Depression stratifications on individual executive functioning tasks. Df= degrees of freedom; F= F-statistic; P-Value= significance level of test. *p < .05, **p < .01, ***p < .001

### Flanker Task


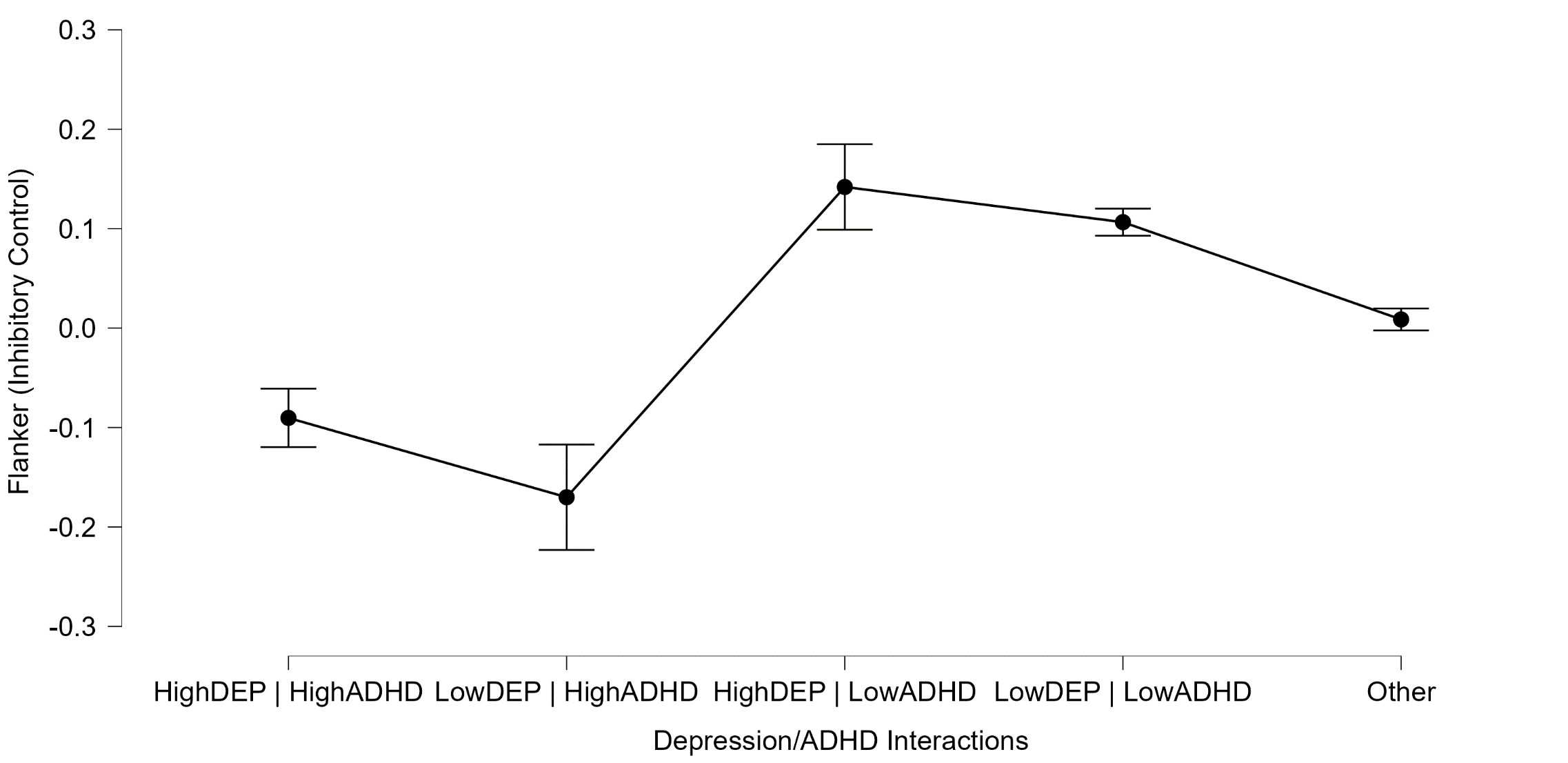


[Supplementary Figure S1: Outcomes of Flanker Task across psychiatric stratifications of the top 15% and lower 15% percentiles of anxiety, depression, and ADHD dimensions. Error bars denote SE.]

| **Post Hoc Comparisons** | | | | | | | | | | | | | | | | | |
| --- | --- | --- | --- | --- | --- | --- | --- | --- | --- | --- | --- | --- | --- | --- | --- | --- | --- |
|  | | | | | | **95% CI for Mean Difference** | | | |  | | | | | | | |
|  | |  | | **Mean Difference** | | **Lower** | | **Upper** | | **SE** | | **t** | | **p_tukey_** | | **p_bonf_** | |
| HighDEP \| HighADHD |  | LowDEP \| HighADHD |  | 0.129 |  | -0.035 |  | 0.293 |  | 0.060 |  | 2.141 |  | 0.203 |  | 0.323 |  |
|  |  | HighDEP \| LowADHD |  | -0.161 |  | -0.308 |  | -0.014 |  | 0.054 |  | -2.979 |  | 0.024 | * | 0.029 | * |
|  |  | LowDEP \| LowADHD |  | -0.135 |  | -0.225 |  | -0.044 |  | 0.033 |  | -4.051 |  | < .001 | *** | < .001 | *** |
|  |  | Other |  | -0.045 |  | -0.133 |  | 0.043 |  | 0.032 |  | -1.401 |  | 0.627 |  | 1.000 |  |
| LowDEP \| HighADHD |  | HighDEP \| LowADHD |  | -0.290 |  | -0.477 |  | -0.103 |  | 0.069 |  | -4.225 |  | < .001 | *** | < .001 | *** |
|  |  | LowDEP \| LowADHD |  | -0.264 |  | -0.410 |  | -0.117 |  | 0.054 |  | -4.899 |  | < .001 | *** | < .001 | *** |
|  |  | Other |  | -0.174 |  | -0.320 |  | -0.029 |  | 0.053 |  | -3.272 |  | 0.009 | ** | 0.011 | * |
| HighDEP \| LowADHD |  | LowDEP \| LowADHD |  | 0.026 |  | -0.101 |  | 0.153 |  | 0.046 |  | 0.561 |  | 0.981 |  | 1.000 |  |
|  |  | Other |  | 0.115 |  | -0.010 |  | 0.240 |  | 0.046 |  | 2.515 |  | 0.087 |  | 0.119 |  |
| LowDEP \| LowADHD |  | Other |  | 0.089 |  | 0.042 |  | 0.137 |  | 0.017 |  | 5.154 |  | < .001 | *** | < .001 | *** |
|  | | | | | | | | | | | | | | | | | |
| * p < .05, ** p < .01, *** p < .001 | | | | | | | | | | | | | | | | | |

[Supplementary Table 2. Tukey’s post-hoc tests assessing group differences in Depression/ADHD psychopathological stratifications on Flanker Task. Mean Difference = the mean difference between groups; Lower = lower bound 95% confidence interval; Upper = upper bound 95% confidence interval; SE = standard error; Ptukey = p-value of given post-hoc test (uncorrected for multiple comparisons); Pbonf = p-value of given post-hoc test (corrected for multiple comparisons). *p < .05, **p < .01, ***p < .001.]

### Card Sorting Task


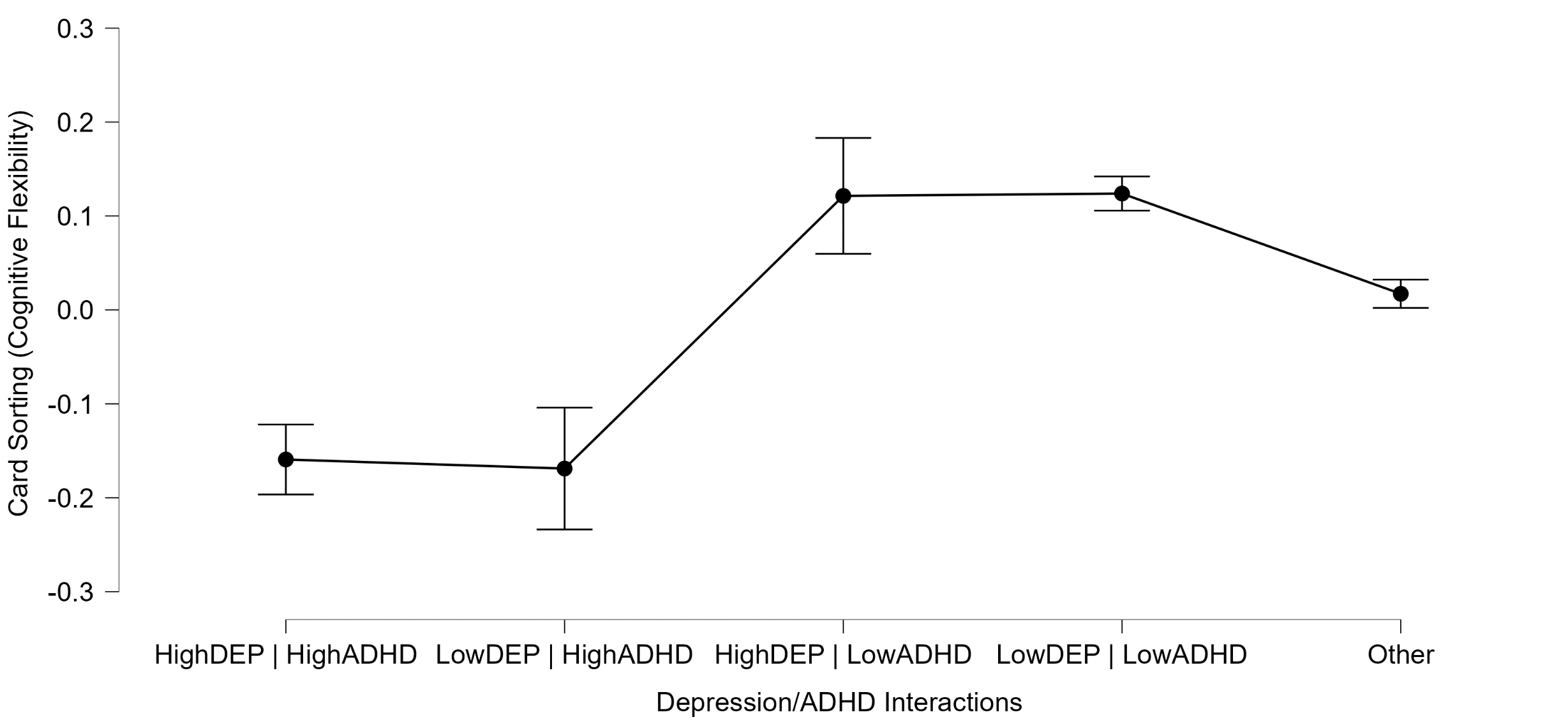


[Supplementary Figure S2: Outcomes of Card Sorting Task across psychiatric stratifications of the top 15% and lower 15% percentiles of anxiety, depression, and ADHD dimensions. Error bars denote SE.]

| **Post Hoc Comparisons** | | | | | | | | | | | | | | | | | |
| --- | --- | --- | --- | --- | --- | --- | --- | --- | --- | --- | --- | --- | --- | --- | --- | --- | --- |
|  | | | | | | **95% CI for Mean Difference** | | | |  | | | | | | | |
|  | |  | | **Mean Difference** | | **Lower** | | **Upper** | | **SE** | | **t** | | **p_tukey_** | | **p_bonf_** | |
| HighDEP \| HighADHD |  | LowDEP \| HighADHD |  | 0.050 |  | -0.177 |  | 0.276 |  | 0.083 |  | 0.598 |  | 0.975 |  | 1.000 |  |
|  |  | HighDEP \| LowADHD |  | -0.159 |  | -0.357 |  | 0.038 |  | 0.072 |  | -2.206 |  | 0.177 |  | 0.274 |  |
|  |  | LowDEP \| LowADHD |  | -0.168 |  | -0.289 |  | -0.046 |  | 0.044 |  | -3.769 |  | 0.002 | ** | 0.002 | ** |
|  |  | Other |  | -0.079 |  | -0.198 |  | 0.040 |  | 0.044 |  | -1.812 |  | 0.366 |  | 0.700 |  |
| LowDEP \| HighADHD |  | HighDEP \| LowADHD |  | -0.209 |  | -0.465 |  | 0.046 |  | 0.094 |  | -2.233 |  | 0.168 |  | 0.256 |  |
|  |  | LowDEP \| LowADHD |  | -0.217 |  | -0.420 |  | -0.014 |  | 0.074 |  | -2.923 |  | 0.029 | * | 0.035 | * |
|  |  | Other |  | -0.129 |  | -0.330 |  | 0.073 |  | 0.074 |  | -1.743 |  | 0.408 |  | 0.814 |  |
| HighDEP \| LowADHD |  | LowDEP \| LowADHD |  | -0.008 |  | -0.177 |  | 0.161 |  | 0.062 |  | -0.133 |  | 1.000 |  | 1.000 |  |
|  |  | Other |  | 0.080 |  | -0.087 |  | 0.248 |  | 0.061 |  | 1.307 |  | 0.687 |  | 1.000 |  |
| LowDEP \| LowADHD |  | Other |  | 0.089 |  | 0.025 |  | 0.152 |  | 0.023 |  | 3.829 |  | 0.001 | ** | 0.001 | ** |
|  | | | | | | | | | | | | | | | | | |
| * p < .05, ** p < .01 | | | | | | | | | | | | | | | | | |
| [Supplementary Table S3. Post-hoc tests assessing group differences in Depression/ADHD psychopathological stratifications on Card Sorting Task.] | | | | | | | | | | | | | | | | | |

### List Sorting Task


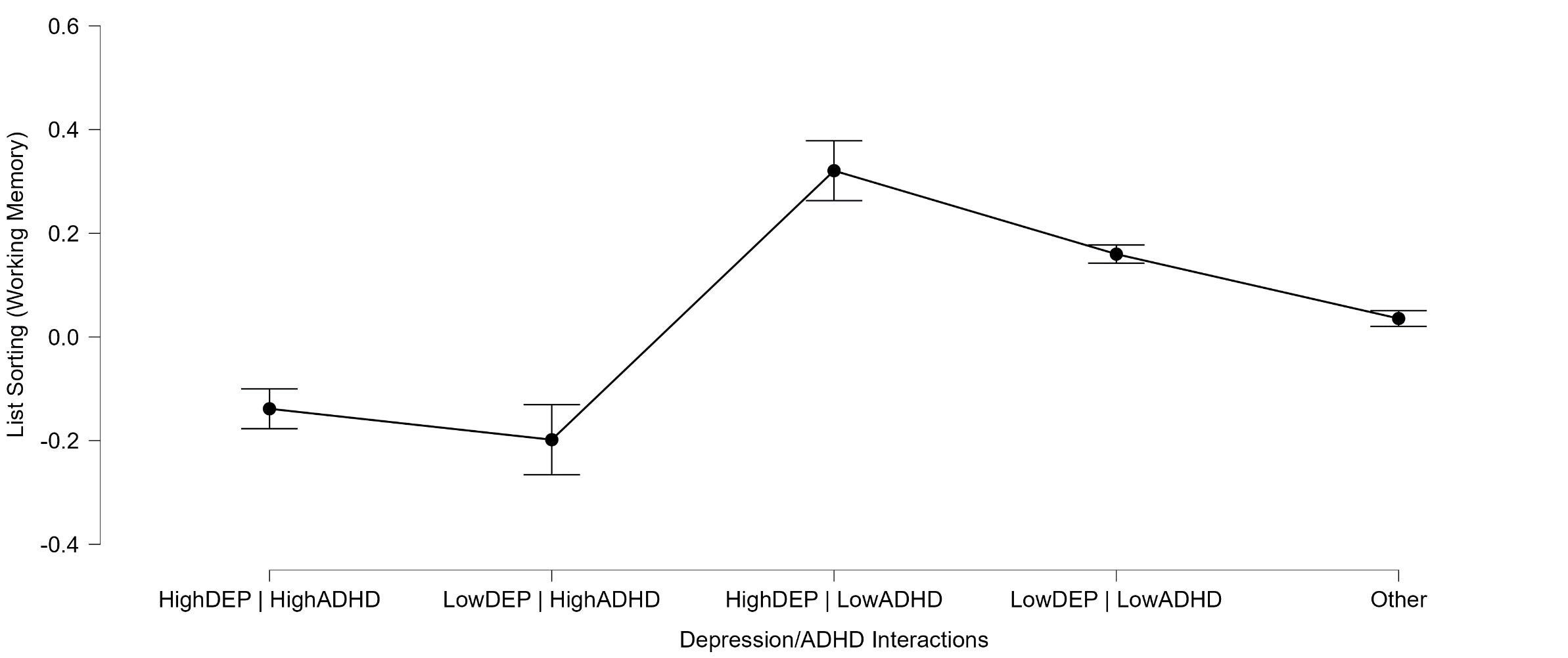


[Supplementary Figure S3: Outcomes of List Sorting Task across psychiatric stratifications of the top 15% and lower 15% percentiles of anxiety, depression, and ADHD dimensions. Error bars denote SE.]

| **Post Hoc Comparisons** | | | | | | | | | | | | | | | | | |
| --- | --- | --- | --- | --- | --- | --- | --- | --- | --- | --- | --- | --- | --- | --- | --- | --- | --- |
|  | | | | | | **95% CI for Mean Difference** | | | |  | | | | | | | |
|  | |  | | **Mean Difference** | | **Lower** | | **Upper** | | **SE** | | **t** | | **p_tukey_** | | **p_bonf_** | |
| HighDEP \| HighADHD |  | LowDEP \| HighADHD |  | 0.094 |  | -0.124 |  | 0.312 |  | 0.080 |  | 1.177 |  | 0.765 |  | 1.000 |  |
|  |  | HighDEP \| LowADHD |  | -0.322 |  | -0.512 |  | -0.131 |  | 0.070 |  | -4.613 |  | < .001 | *** | < .001 | *** |
|  |  | LowDEP \| LowADHD |  | -0.170 |  | -0.287 |  | -0.053 |  | 0.043 |  | -3.962 |  | < .001 | *** | < .001 | *** |
|  |  | Other |  | -0.073 |  | -0.188 |  | 0.042 |  | 0.042 |  | -1.728 |  | 0.417 |  | 0.841 |  |
| LowDEP \| HighADHD |  | HighDEP \| LowADHD |  | -0.416 |  | -0.662 |  | -0.170 |  | 0.090 |  | -4.610 |  | < .001 | *** | < .001 | *** |
|  |  | LowDEP \| LowADHD |  | -0.264 |  | -0.459 |  | -0.069 |  | 0.072 |  | -3.695 |  | 0.002 | ** | 0.002 | ** |
|  |  | Other |  | -0.167 |  | -0.361 |  | 0.027 |  | 0.071 |  | -2.348 |  | 0.130 |  | 0.189 |  |
| HighDEP \| LowADHD |  | LowDEP \| LowADHD |  | 0.151 |  | -0.012 |  | 0.314 |  | 0.060 |  | 2.535 |  | 0.083 |  | 0.113 |  |
|  |  | Other |  | 0.249 |  | 0.087 |  | 0.410 |  | 0.059 |  | 4.198 |  | < .001 | *** | < .001 | *** |
| LowDEP \| LowADHD |  | Other |  | 0.097 |  | 0.036 |  | 0.158 |  | 0.022 |  | 4.358 |  | < .001 | *** | < .001 | *** |
|  | | | | | | | | | | | | | | | | | |
| * p < .05, ** p < .01, *** p < .001 | | | | | | | | | | | | | | | | | |
| [Supplementary Table S4. Post-hoc tests assessing group differences in Depression/ADHD psychopathological stratifications on List Sorting Task.] | | | | | | | | | | | | | | | | | |
|  | | | | | | | | | | | | | | | | | |

## S2: Cognitive Task Differences among Psychiatric Stratifications

**Cognitive Performance Between *HighDEP | LowADHD* and *LowDEP | LowADHD***

| **Independent Samples T-Test** | | | | | | | | | | | | | |
| --- | --- | --- | --- | --- | --- | --- | --- | --- | --- | --- | --- | --- | --- |
|  | | **t** | | **df** | | **p** | | **Mean Difference** | | **SE Difference** | | **Cohen’s d** | |
| Picture Vocabulary |  | 6.015 |  | 749.695 |  | < .001 |  | 0.242 |  | 0.040 |  | 0.247 |  |
| Flanker Inhibitory Control |  | 0.357 |  | 746.056 |  | 0.361 |  | 0.014 |  | 0.040 |  | 0.015 |  |
| List Sorting Working Memory |  | 2.157 |  | 446.702 |  | 0.016 |  | 0.114 |  | 0.053 |  | 0.115 |  |
| Dimensional Card Sorting |  | 0.364 |  | 446.079 |  | 0.358 |  | 0.020 |  | 0.055 |  | 0.020 |  |
| Pattern Comparison Processing Speed |  | 0.963 |  | 725.640 |  | 0.168 |  | 0.040 |  | 0.042 |  | 0.041 |  |
| Picture Sequence Memory |  | -0.492 |  | 747.182 |  | 0.689 |  | -0.020 |  | 0.041 |  | -0.020 |  |
| Oral Reading Recognition |  | 4.022 |  | 735.690 |  | < .001 |  | 0.170 |  | 0.042 |  | 0.169 |  |
| Rey Auditory Verbal Learning (RAVLT) |  | 2.011 |  | 728.119 |  | 0.022 |  | 0.080 |  | 0.040 |  | 0.084 |  |
| ravlt_l_repitition |  | -0.655 |  | 715.483 |  | 0.744 |  | -0.046 |  | 0.070 |  | -0.028 |  |
| ravlt_l_intrusions |  | -1.543 |  | 770.033 |  | 0.938 |  | -0.046 |  | 0.030 |  | -0.061 |  |
| Matrix Reasoning |  | 1.508 |  | 436.583 |  | 0.066 |  | 0.078 |  | 0.052 |  | 0.081 |  |
| Emotional N Back_accuracy_positive |  | 0.157 |  | 667.938 |  | 0.438 |  | 0.007 |  | 0.042 |  | 0.007 |  |
| Emotional N Back_accuracy_neg |  | 0.366 |  | 644.728 |  | 0.357 |  | 0.017 |  | 0.046 |  | 0.017 |  |
| Little Man Task_correct |  | 0.847 |  | 731.985 |  | 0.199 |  | 0.215 |  | 0.254 |  | 0.036 |  |
| Little Man Task_correct_reactiontime |  | -0.312 |  | 739.404 |  | 0.622 |  | -0.012 |  | 0.040 |  | -0.013 |  |
| Stroop accuracy incongruent |  | 1.050 |  | 790.580 |  | 0.147 |  | 0.038 |  | 0.036 |  | 0.044 |  |
| Stroop reactiontime incongruent |  | 0.159 |  | 769.646 |  | 0.437 |  | 0.007 |  | 0.042 |  | 0.007 |  |
| correctRT Arithmetic |  | -1.295 |  | 146.800 |  | 0.901 |  | -0.110 |  | 0.085 |  | -0.120 |  |
| Totalcorrect Arithmetic |  | 2.768 |  | 151.043 |  | 0.003 |  | 5.634 |  | 2.036 |  | 0.253 |  |
| Totalcorrect Enumeration |  | 2.023 |  | 155.741 |  | 0.022 |  | 1.199 |  | 0.593 |  | 0.179 |  |
|  | | | | | | | | | | | | | |
| *Note.*  For all tests, the alternative hypothesis specifies that group *HighDEP \| LowADHD* is greater than group *LowDEP \| LowADHD* . | | | | | | | | | | | | | |
| *Note.*  Welch’s t-test. | | | | | | | | | | | | | |

[Supplementary Table S5: Comparison of High Depression|LowADHD to Low Depression|Low ADHD across task battery. Indicates the dimension of depression does not impair cognitive performance in adolescents if they have no co-occurring ADHD, and moderately outperform individuals low in both dimensions on some tasks.]

**Cognitive Performance Between *LowDEP | HighADHD* and *HighDEP | LowADHD***

| **Independent Samples T-Test** | | | | | | | | | | | | | |
| --- | --- | --- | --- | --- | --- | --- | --- | --- | --- | --- | --- | --- | --- |
|  | | **t** | | **df** | | **p** | | **Mean Difference** | | **SE Difference** | | **Cohen’s d** | |
| Picture Vocabulary |  | -6.906 |  | 1013.172 |  | < .001 |  | -0.414 |  | 0.060 |  | -0.419 |  |
| Flanker Inhibitory Control |  | -4.619 |  | 984.710 |  | < .001 |  | -0.283 |  | 0.061 |  | -0.282 |  |
| List Sorting Working Memory |  | -6.501 |  | 628.489 |  | < .001 |  | -0.509 |  | 0.078 |  | -0.504 |  |
| Dimensional Card Sorting |  | -4.182 |  | 664.437 |  | < .001 |  | -0.320 |  | 0.077 |  | -0.321 |  |
| Pattern Comparison Processing Speed |  | -5.677 |  | 1036.522 |  | < .001 |  | -0.340 |  | 0.060 |  | -0.345 |  |
| Picture Sequence Memory |  | -5.484 |  | 1073.208 |  | < .001 |  | -0.316 |  | 0.058 |  | -0.330 |  |
| Oral Reading Recognition |  | -8.424 |  | 1062.975 |  | < .001 |  | -0.505 |  | 0.060 |  | -0.508 |  |
| Rey Auditory Verbal Learning (RAVLT) |  | -6.629 |  | 963.611 |  | < .001 |  | -0.410 |  | 0.062 |  | -0.407 |  |
| ravlt_l_repitition |  | 1.072 |  | 970.554 |  | 0.858 |  | 0.117 |  | 0.109 |  | 0.066 |  |
| ravlt_l_intrusions |  | 4.098 |  | 852.820 |  | 1.000 |  | 0.213 |  | 0.052 |  | 0.253 |  |
| Matrix Reasoning |  | -4.385 |  | 568.969 |  | < .001 |  | -0.364 |  | 0.083 |  | -0.346 |  |
| Emotional N Back_accuracy_positive |  | -4.900 |  | 887.569 |  | < .001 |  | -0.314 |  | 0.064 |  | -0.315 |  |
| Emotional N Back_accuracy_neg |  | -4.929 |  | 951.718 |  | < .001 |  | -0.318 |  | 0.065 |  | -0.315 |  |
| Little Man Task_correct |  | -4.721 |  | 1019.319 |  | < .001 |  | -1.771 |  | 0.375 |  | -0.287 |  |
| Little Man Task_correct_reactiontime |  | -2.334 |  | 980.350 |  | 0.010 |  | -0.143 |  | 0.061 |  | -0.143 |  |
| Stroop_accuracy_incongruent |  | -6.303 |  | 765.175 |  | < .001 |  | -0.408 |  | 0.065 |  | -0.401 |  |
| Stroop_reactiontime_incongruent |  | 4.291 |  | 952.963 |  | 1.000 |  | 0.268 |  | 0.063 |  | 0.266 |  |
| correctRT Arithmetic |  | 3.407 |  | 173.938 |  | 1.000 |  | 0.486 |  | 0.143 |  | 0.474 |  |
| Totalcorrect Arithmetic |  | -5.460 |  | 195.123 |  | < .001 |  | -16.733 |  | 3.064 |  | -0.749 |  |
| Totalcorrect Enumeration |  | -4.756 |  | 181.996 |  | < .001 |  | -4.521 |  | 0.950 |  | -0.656 |  |
|  | | | | | | | | | | | | | |
| *Note.*  For all tests, the alternative hypothesis specifies that group *LowDEP \| HighADHD* is less than group *HighDEP \| LowADHD* . | | | | | | | | | | | | | |
| *Note.*  Welch’s t-test. | | | | | | | | | | | | | |

[Supplementary Table S6: Comparison of High Depression|LowADHD to Low Depression|High ADHD across task battery. Indicates the dimension of ADHD compared to depression is strongly affecting task performance.]

## S3: Total Cognitive Performance as a function of trait worry


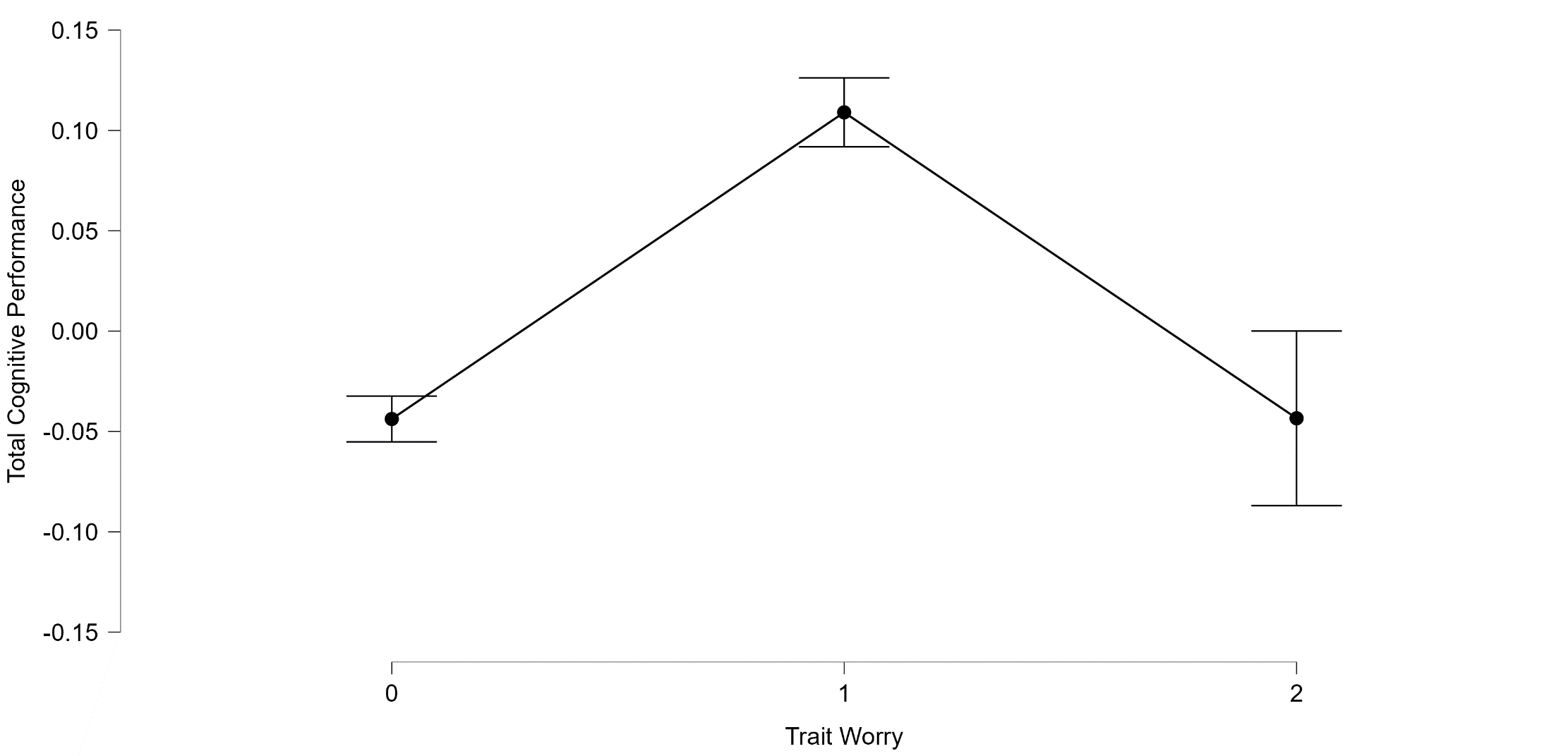


[Supplementary Figure S4: Outcomes of Total Cognitive Performance as a function of trait worry. Error Bars denote SEM]

A one-way ANOVA showed statistically significant differences in magnitude of worry on total cognitive performance. Specifically, those with moderate amounts of worry (.12 ± 1) outperformed those with low (-.04 ± 1) and high (-.04 ± 1) amounts of worry (F(2, 11536) = [27.699], p <.001) in overall task performance.

| **ANOVA – Total Cognitive Performance** | | | | | | | | | | | |
| --- | --- | --- | --- | --- | --- | --- | --- | --- | --- | --- | --- |
| **Cases** | | **Sum of Squares** | | **df** | | **Mean Square** | | **F** | | **p** | |
| worries |  | 55.159 |  | 2 |  | 27.580 |  | 27.699 |  | < .001 |  |
| Residuals |  | 11486.331 |  | 11536 |  | 0.996 |  |  |  |  |  |
|  | | | | | | | | | | | |
| *Note.*  Type III Sum of Squares | | | | | | | | | | | |

## S4: Total Cognitive Performance as a function of trait of perfectionism


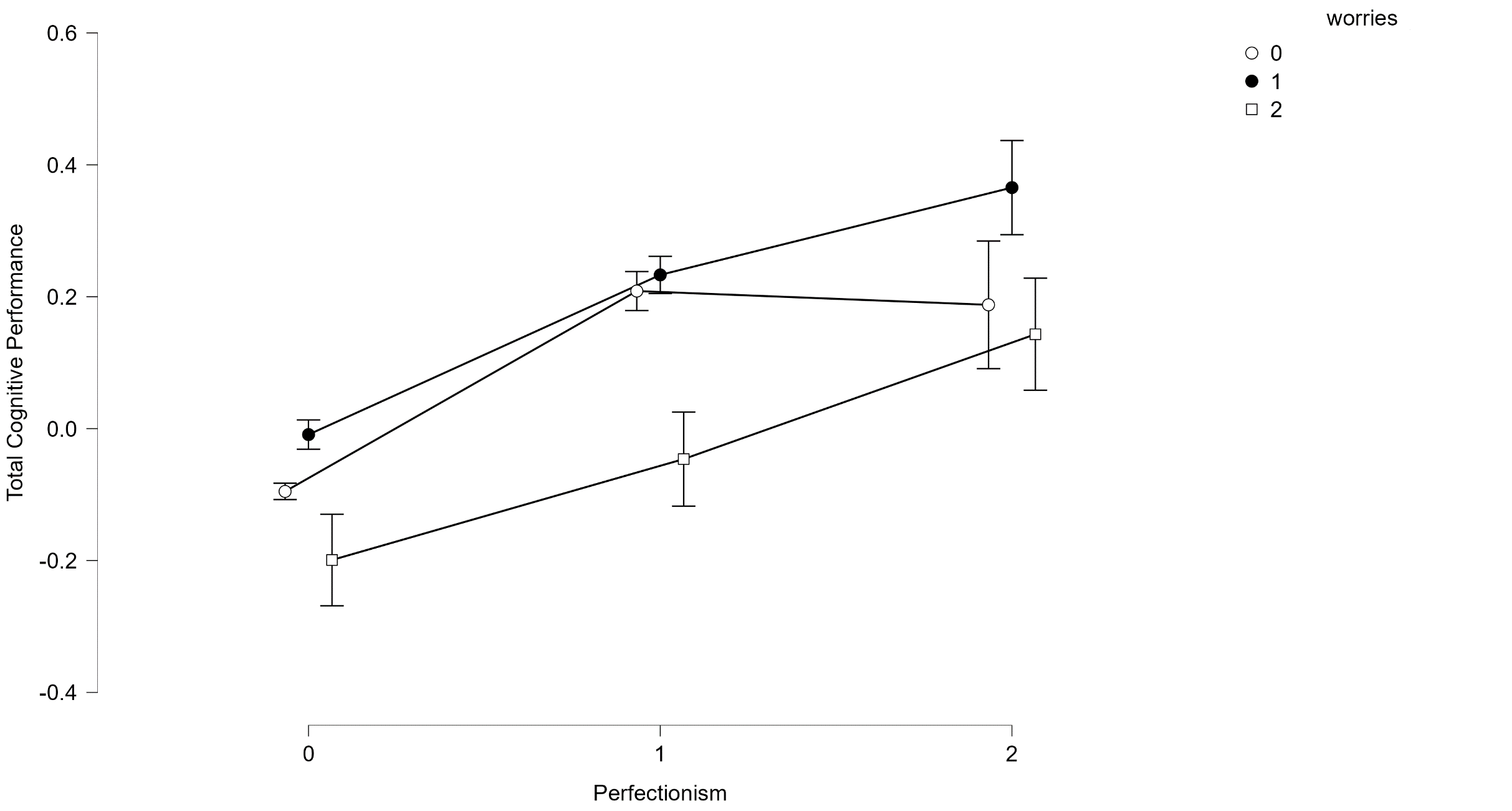


[Supplementary Figure S5: Total Cognitive Performance as a function of trait perfectionism. Different lines for trait worry. Error Bars denote SEM.]

A two-way ANOVA revealed significant main effects of worry (F(2, 11530) = [10.264], p <.001) and perfectionism (F(2, 11530) = [27.055], p <.001) on total cognitive performance. Importantly, those with moderate levels of worry together with high levels of perfectionism demonstrated superior task performance compared to all other combinations of worry/perfectionism stratifications. No significant interaction effect was found between worrying and perfectionism traits on total cognitive performance (p > .05).

| **ANOVA – Total Cognitive Performance** | | | | | | | | | | | |
| --- | --- | --- | --- | --- | --- | --- | --- | --- | --- | --- | --- |
| **Cases** | | **Sum of Squares** | | **df** | | **Mean Square** | | **F** | | **p** | |
| worries |  | 20.153 |  | 2 |  | 10.077 |  | 10.264 |  | < .001 |  |
| perfectionist |  | 53.120 |  | 2 |  | 26.560 |  | 27.055 |  | < .001 |  |
| worries ✻ perfectionist |  | 4.068 |  | 4 |  | 1.017 |  | 1.036 |  | 0.387 |  |
| Residuals |  | 11319.203 |  | 11530 |  | 0.982 |  |  |  |  |  |
|  | | | | | | | | | | | |
| *Note.*  Type III Sum of Squares | | | | | | | | | | | |

## S5: Left: Fluid Intelligence differences in MDD diagnosis. Right: Fluid Intelligence in MDD diagnosis as a function of concentration problems.


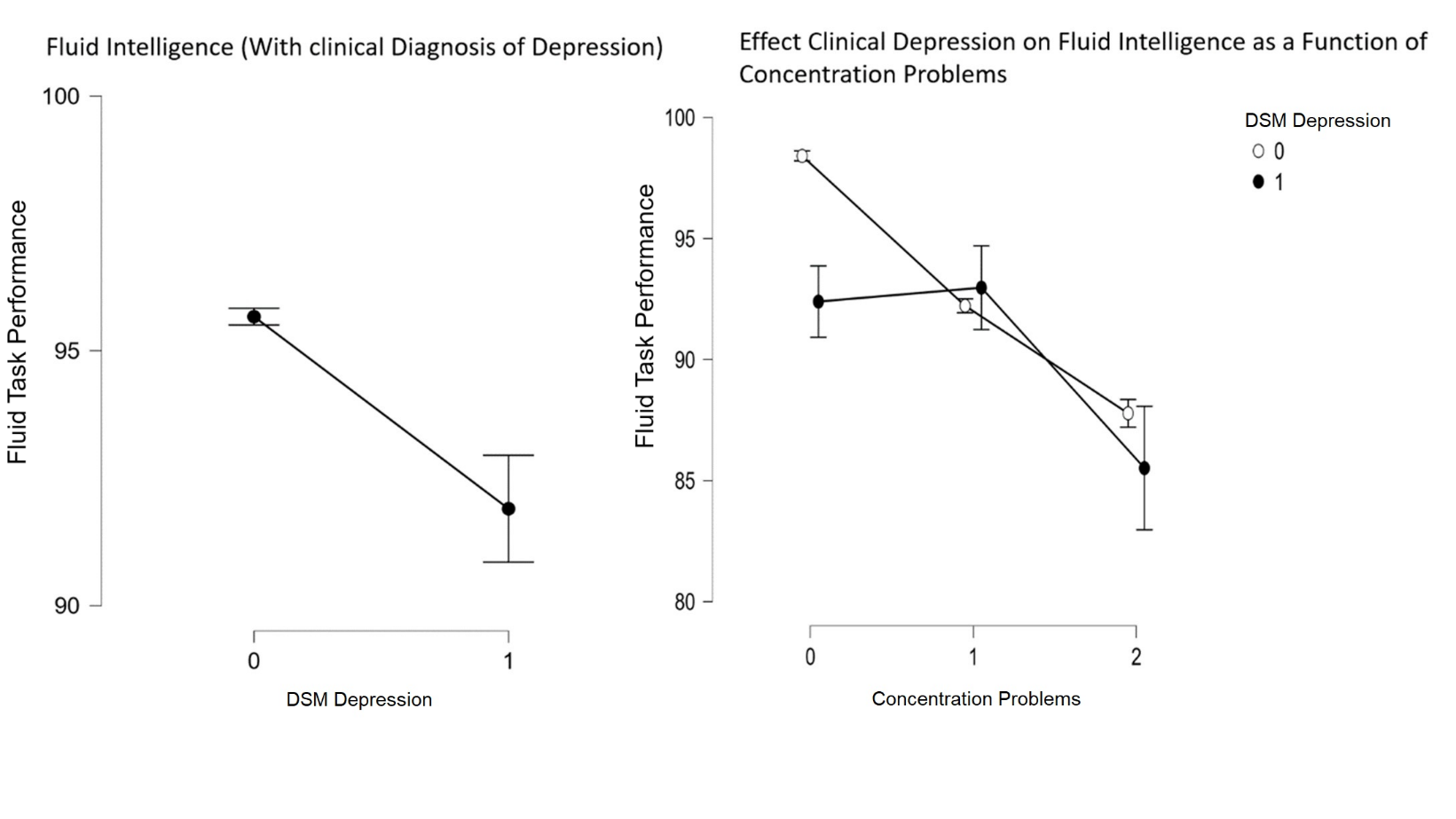


[Supplementary Figure S6: Error Bars denote SEM]

As expected, a one-way ANOVA showed that those with low levels of depression (95.7 ± 17.3) outperformed those high levels of depression (91.9 ± 17.3) in measures of fluid cognitive performance (F(1, 11483) = [12.99], p <.001).

| **ANOVA – Fluid Task Performance** | | | | | | | | | | | |
| --- | --- | --- | --- | --- | --- | --- | --- | --- | --- | --- | --- |
| **Cases** | | **Sum of Squares** | | **df** | | **Mean Square** | | **F** | | **p** | |
| Depressed clinical |  | 3909.504 |  | 1 |  | 3909.504 |  | 12.986 |  | < .001 |  |
| Residuals |  | 3.457e+6 |  | 11483 |  | 301.062 |  |  |  |  |  |
|  | | | | | | | | | | | |
| *Note.*  Type III Sum of Squares | | | | | | | | | | | |

However, when concentration difficulty was included in the ANOVA model (i.e., instantiation of a two-way ANOVA), a significant interaction effect was found with depressive symptoms and concentration difficulties on fluid cognitive performance (F(2, 11472) = [4.841], p =.008); with those with high depression and moderate concentration problems (92.39 ± 16.6) outperforming those with low depression and moderate concentration problems (98.41 ± 17), and those with high depression and high concentration problems (85.52 ± 14.2) underperforming in comparison to those who indicate low depression and high concentration problems (87.8 ± 17.32).

| **ANOVA – Fluid Task Performance** | | | | | | | | | | | |
| --- | --- | --- | --- | --- | --- | --- | --- | --- | --- | --- | --- |
| **Cases** | | **Sum of Squares** | | **df** | | **Mean Square** | | **F** | | **p** | |
| Depressed clinical |  | 1139.566 |  | 1 |  | 1139.566 |  | 3.956 |  | 0.047 |  |
| Concentration problems |  | 7745.298 |  | 2 |  | 3872.649 |  | 13.443 |  | < .001 |  |
| Depressed clinical ✻ Concentration problems |  | 2789.248 |  | 2 |  | 1394.624 |  | 4.841 |  | 0.008 |  |
| Residuals |  | 3.305e+6 |  | 11472 |  | 288.077 |  |  |  |  |  |
|  | | | | | | | | | | | |
| *Note.*  Type III Sum of Squares | | | | | | | | | | | |

## S6: Left: School performance differences in MDD diagnosis. Right: School performance differences in MDD diagnosis as a function of concentration problems.


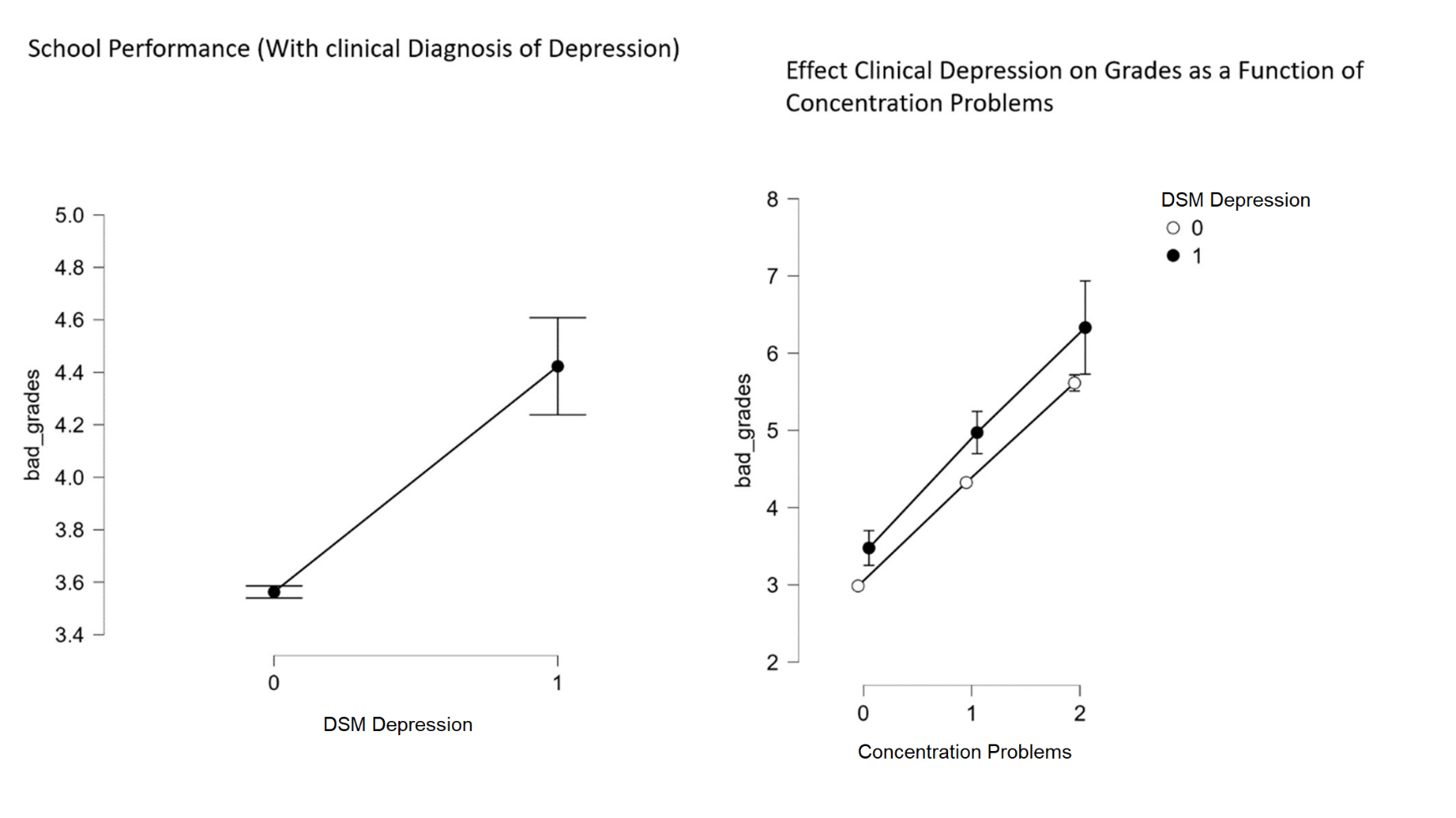


[Supplementary Figure S7: Error Bars denote SEM]

Likewise, a one-way ANOVA showed that those with low levels of depression (3.56 ± 2.3) outperformed those high levels of depression (4.4 ± 2.62) in overall school performance (F(1, 10206) = [27.37], p <.001).

| **ANOVA – bad grades** | | | | | | | | | | | |
| --- | --- | --- | --- | --- | --- | --- | --- | --- | --- | --- | --- |
| **Cases** | | **Sum of Squares** | | **df** | | **Mean Square** | | **F** | | **p** | |
| Depressed clinical |  | 145.759 |  | 1 |  | 145.759 |  | 27.370 |  | < .001 |  |
| Residuals |  | 54351.331 |  | 10206 |  | 5.325 |  |  |  |  |  |
|  | | | | | | | | | | | |
| *Note.*  Type III Sum of Squares | | | | | | | | | | | |

When concentration difficulty was included in the ANOVA model, both depressed mood (F(1, 10198) = [11.89], p <.001)) and concentration problems (F(2, 10198) = [76.62], p <.001)) showed significant main effects (but no significant interaction effect) on school performance; with those with high depression performing worse than those with low depression, no matter the level of concentration difficulty.

| **ANOVA – bad grades** | | | | | | | | | | | |
| --- | --- | --- | --- | --- | --- | --- | --- | --- | --- | --- | --- |
| **Cases** | | **Sum of Squares** | | **df** | | **Mean Square** | | **F** | | **p** | |
| Depressed clinical |  | 55.192 |  | 1 |  | 55.192 |  | 11.888 |  | < .001 |  |
| Concentration problems |  | 739.292 |  | 2 |  | 369.646 |  | 79.619 |  | < .001 |  |
| Depressed clinical ✻ Concentration problems |  | 1.566 |  | 2 |  | 0.783 |  | 0.169 |  | 0.845 |  |
| Residuals |  | 47346.065 |  | 10198 |  | 4.643 |  |  |  |  |  |
|  | | | | | | | | | | | |
| *Note.*  Type III Sum of Squares | | | | | | | | | | | |

## S7: EF and Psychopathological Differences among Psychiatric Stratifications

**Differences in other Psychopathological Features, Parent-Rated Executive Function, and Behavioral Inhibition Scales Between *HighDEP | HighADHD* and *HighDEP | LowADHD***

| **Independent Samples T-Test** | | | | | | | | | |
| --- | --- | --- | --- | --- | --- | --- | --- | --- | --- |
|  | | **t** | | **df** | | **p** | | **Cohen’s d** | |
| aggressive |  | 49.400 |  | 4359.077 |  | < .001 |  | 1.397 |  |
| argues |  | 34.308 |  | 2787.232 |  | < .001 |  | 1.103 |  |
| obsessions |  | 32.761 |  | 3150.202 |  | < .001 |  | 1.025 |  |
| worries |  | 7.301 |  | 2596.548 |  | < .001 |  | 0.238 |  |
| lonely |  | 15.101 |  | 3128.209 |  | < .001 |  | 0.473 |  |
| selfharm |  | 3.017 |  | 3069.114 |  | 0.001 |  | 0.095 |  |
| disobedient_home |  | 33.059 |  | 2997.303 |  | < .001 |  | 1.046 |  |
| disobedient_school |  | 34.020 |  | 4353.450 |  | < .001 |  | 0.943 |  |
| no_guilt |  | 26.132 |  | 4114.252 |  | < .001 |  | 0.763 |  |
| breaks_rules |  | 37.691 |  | 3933.123 |  | < .001 |  | 1.117 |  |
| nervous_twitching |  | 24.129 |  | 4190.220 |  | < .001 |  | 0.700 |  |
| unknown_physical_problems |  | 3.312 |  | 2863.177 |  | < .001 |  | 0.106 |  |
| compulsions |  | 24.253 |  | 4368.449 |  | < .001 |  | 0.676 |  |
| screams |  | 24.254 |  | 4108.719 |  | < .001 |  | 0.709 |  |
| speech_problems |  | 9.110 |  | 3555.663 |  | < .001 |  | 0.277 |  |
| suicidal |  | 6.853 |  | 3348.634 |  | < .001 |  | 0.212 |  |
| difficulty_making_friends |  | 10.446 |  | 1347.574 |  | < .001 |  | 0.540 |  |
| regarded_weird |  | 15.850 |  | 1419.232 |  | < .001 |  | 0.806 |  |
| narrow_interests |  | 10.872 |  | 1342.530 |  | < .001 |  | 0.563 |  |
| bdefs_calm_down |  | 9.605 |  | 363.793 |  | < .001 |  | 0.814 |  |
| bdefs_consequences |  | 20.798 |  | 567.627 |  | < .001 |  | 1.579 |  |
| bdefs_distract_upset |  | 8.964 |  | 364.908 |  | < .001 |  | 0.760 |  |
| bdefs_explain_idea |  | 12.612 |  | 396.755 |  | < .001 |  | 1.048 |  |
| bdefs_explain_pt |  | 12.914 |  | 487.469 |  | < .001 |  | 1.021 |  |
| bdefs_explain_seq |  | 15.053 |  | 600.912 |  | < .001 |  | 1.124 |  |
| bdefs_impulsive_action |  | 20.251 |  | 547.088 |  | < .001 |  | 1.554 |  |
| bdefs_inconsistant |  | 15.284 |  | 359.920 |  | < .001 |  | 1.301 |  |
| bdefs_lazy |  | 9.743 |  | 327.894 |  | < .001 |  | 0.848 |  |
| bdefs_process_info |  | 15.301 |  | 630.787 |  | < .001 |  | 1.119 |  |
| bdefs_rechannel |  | 9.138 |  | 354.446 |  | < .001 |  | 0.780 |  |
| bdefs_sense_time |  | 15.225 |  | 293.491 |  | < .001 |  | 1.365 |  |
| bdefs_shortcuts |  | 13.385 |  | 330.694 |  | < .001 |  | 1.164 |  |
| bdefs_stop_think |  | 17.748 |  | 497.899 |  | < .001 |  | 1.394 |  |
| up_negative_urgency |  | 8.080 |  | 1313.088 |  | < .001 |  | 0.368 |  |
| up_lackofplanning |  | 11.320 |  | 1481.609 |  | < .001 |  | 0.502 |  |
| up_sensationseeking |  | 2.851 |  | 1214.874 |  | 0.002 |  | 0.132 |  |
| up_positiveurgency |  | 10.660 |  | 1367.974 |  | < .001 |  | 0.481 |  |
| up_lackperseverance |  | 13.170 |  | 1542.955 |  | < .001 |  | 0.579 |  |
| bis_behav_inhibition |  | -2.016 |  | 1248.369 |  | 0.978 |  | -0.093 |  |
| bis_reward_responsive |  | 3.013 |  | 1249.695 |  | 0.001 |  | 0.139 |  |
| bis_drive |  | 8.134 |  | 1363.673 |  | < .001 |  | 0.368 |  |
| bis_funseeking |  | 7.776 |  | 1316.602 |  | < .001 |  | 0.354 |  |
| difficulty_goingtosleep |  | 6.709 |  | 2773.654 |  | < .001 |  | 0.216 |  |
| difficulty_wakingup |  | 15.820 |  | 2983.609 |  | < .001 |  | 0.501 |  |
| daytime_sleepiness |  | 10.113 |  | 2897.791 |  | < .001 |  | 0.322 |  |
| demands_attention |  | 33.740 |  | 3791.624 |  | < .001 |  | 1.011 |  |
| avoids_eyecontact |  | 14.004 |  | 1416.548 |  | < .001 |  | 0.712 |  |
| bad_conversational_flow |  | 19.247 |  | 1437.489 |  | < .001 |  | 0.930 |  |
| sensory_sensitivity |  | 10.600 |  | 1314.808 |  | < .001 |  | 0.551 |  |
| concentration_on_parts |  | 19.756 |  | 1472.518 |  | < .001 |  | 0.982 |  |
|  | | | | | | | | | |
| *Note.*  For all tests, the alternative hypothesis specifies that group *HighDEP \| HighADHD* is greater than group *HighDEP \| LowADHD* . | | | | | | | | | |
| *Note.*  Welch’s t-test. | | | | | | | | | |

[Supplementary Table S7: Subsequent exploratory analysis of co-occurring symptoms of ADHD and depression compared to depressive symptoms alone on other psychopathological features, parent-rated executive functioning from the Barkleys scale (BDEFS), Behavioural Inhibition System (BIS), Behavioral Inhibition Scale (UPPS), and Sleep Problems. Indicates that co-occurring ADHD and Depression dimensions exacerbate other psychopathological features (especially disobedience/aggressive tendencies, negative peer relationships, and attention-seeking) and are associated with worse executive functioning, behavioural inhibition, and autism dimensions.]

## S8: Environmental Differences among Psychiatric Stratifications

**Differences in other Environmental Variables and Parent Pathology Scales Between *HighDEP | HighADHD* and *LowDEP | LowADHD***

| **Independent Samples T-Test** | | | | | | | | | |
| --- | --- | --- | --- | --- | --- | --- | --- | --- | --- |
|  | | **t** | | **df** | | **p** | | **Cohen’s d** | |
| father_druguse |  | 6.330 |  | 260.646 |  | < .001 |  | 0.526 |  |
| mother_druguse |  | 3.037 |  | 256.350 |  | 0.001 |  | 0.256 |  |
| birth_weight |  | 0.023 |  | 320.206 |  | 0.491 |  | 0.002 |  |
| parent_suicide |  | 4.728 |  | 257.588 |  | < .001 |  | 0.397 |  |
| mother_depression |  | 9.642 |  | 279.015 |  | < .001 |  | 0.761 |  |
| father_depression |  | 5.504 |  | 275.346 |  | < .001 |  | 0.438 |  |
| frequent_family_conflict |  | 8.660 |  | 263.207 |  | < .001 |  | 0.714 |  |
| family_conflict_ss |  | 12.681 |  | 291.592 |  | < .001 |  | 0.970 |  |
| parent_internalizing |  | 19.582 |  | 283.903 |  | < .001 |  | 1.525 |  |
| parent_externalizing |  | 17.327 |  | 292.936 |  | < .001 |  | 1.321 |  |
| parent_depression |  | 14.647 |  | 249.559 |  | < .001 |  | 1.265 |  |
| parent_anxiety |  | 11.828 |  | 249.869 |  | < .001 |  | 1.021 |  |
| parent_adhd |  | 12.416 |  | 244.454 |  | < .001 |  | 1.093 |  |
| socialmedia_hoursperday |  | 4.152 |  | 299.500 |  | < .001 |  | 0.312 |  |
| adverse_life_events |  | 6.099 |  | 281.024 |  | < .001 |  | 0.479 |  |
|  | | | | | | | | | |
| *Note.*  For all tests, the alternative hypothesis specifies that group *HighDEP \| HighADHD* is greater than group *LowDEP \| LowADHD* . | | | | | | | | | |
| *Note.*  Welch’s t-test. | | | | | | | | | |

[Supplementary Table S8A: Subsequent exploratory analysis of co-occurring symptoms of ADHD and depression (HighDEP | HighADHD) compared to individuals low in both dimensions (LowDEP | LowADHD) associated with a range of adverse environmental variables and parental pathologies.]

| **Independent Samples T-Test** | | | | | | | | | |
| --- | --- | --- | --- | --- | --- | --- | --- | --- | --- |
|  | | **t** | | **df** | | **p** | | **Cohen’s d** | |
| father_druguse |  | 3.446 |  | 1814.561 |  | < .001 |  | 0.150 |  |
| mother_druguse |  | 2.186 |  | 1755.420 |  | 0.014 |  | 0.095 |  |
| birth_weight |  | -4.059 |  | 2008.150 |  | 1.000 |  | -0.175 |  |
| parent_suicide |  | 0.717 |  | 1917.123 |  | 0.237 |  | 0.031 |  |
| mother_depression |  | -0.252 |  | 2017.520 |  | 0.599 |  | -0.011 |  |
| father_depression |  | -1.072 |  | 1980.463 |  | 0.858 |  | -0.047 |  |
| frequent_family_conflict |  | 1.541 |  | 2100.116 |  | 0.062 |  | 0.065 |  |
| family_conflict |  | 1.754 |  | 2111.816 |  | 0.040 |  | 0.074 |  |
| parent_internalizing |  | -7.346 |  | 1094.858 |  | 1.000 |  | -0.438 |  |
| parent_externalizing |  | -0.219 |  | 1060.043 |  | 0.586 |  | -0.013 |  |
| parent_depression |  | -6.837 |  | 1128.639 |  | 1.000 |  | -0.404 |  |
| parent_anxiety |  | -4.415 |  | 1128.084 |  | 1.000 |  | -0.260 |  |
| parent_adhd |  | -0.346 |  | 1062.732 |  | 0.635 |  | -0.021 |  |
| socialmedia_hoursperday |  | 0.871 |  | 386.129 |  | 0.192 |  | 0.084 |  |
| adverse_life_events |  | 0.341 |  | 2111.636 |  | 0.367 |  | 0.014 |  |
| not_liked |  | 1.074 |  | 2097.949 |  | 0.141 |  | 0.045 |  |
|  | | | | | | | | | |
| *Note.*  For all tests, the alternative hypothesis specifies that group *LowDEP \| HighADHD* is greater than group *HighDEP \| LowADHD* . | | | | | | | | | |
| *Note.*  Welch’s t-test. | | | | | | | | | |

[Supplementary Table S8B: Subsequent exploratory analysis of *LowDEP | HighADHD compared to HighDEP | LowADHD* with a range of adverse environmental variables and parental pathologies.]

## S9: GGMs

See Epskamp et al (2018) for estimating network stability.

[Supplementary Figure S9A: Case-Drop Bootstrapped Network for Edges and Centrality scores in Figure 4 Panel A from original MS, nBoots = 1,000. Threshold = .1]

[Supplementary Figure S9B: Case-Drop Bootstrapped Network for Edges and Centrality scores in Figure 4 Panel B from original MS, nBoots = 1,000. Threshold = .1]

| **Summary of Network** | | | | | | | |
| --- | --- | --- | --- | --- | --- | --- | --- |
| **Network** | | **Number of nodes** | | **Number of non-zero edges** | | **Sparsity** | |
| Time 0 |  | 21 |  | 35 / 210 |  | 0.833 |  |
| Time 1 |  | 21 |  | 39 / 210 |  | 0.814 |  |
| Time 2 |  | 21 |  | 38 / 210 |  | 0.819 |  |
| Time 3 |  | 21 |  | 41 / 210 |  | 0.805 |  |
|  | | | | | | | |

[Supplementary Table S9: Number of non-zero edges and sparsity of network from Figure 4 Panel B across time points]

## S10: Larger-Scale Relationships Among Psychopathological Traits and Fluid Intelligence in Adolescence


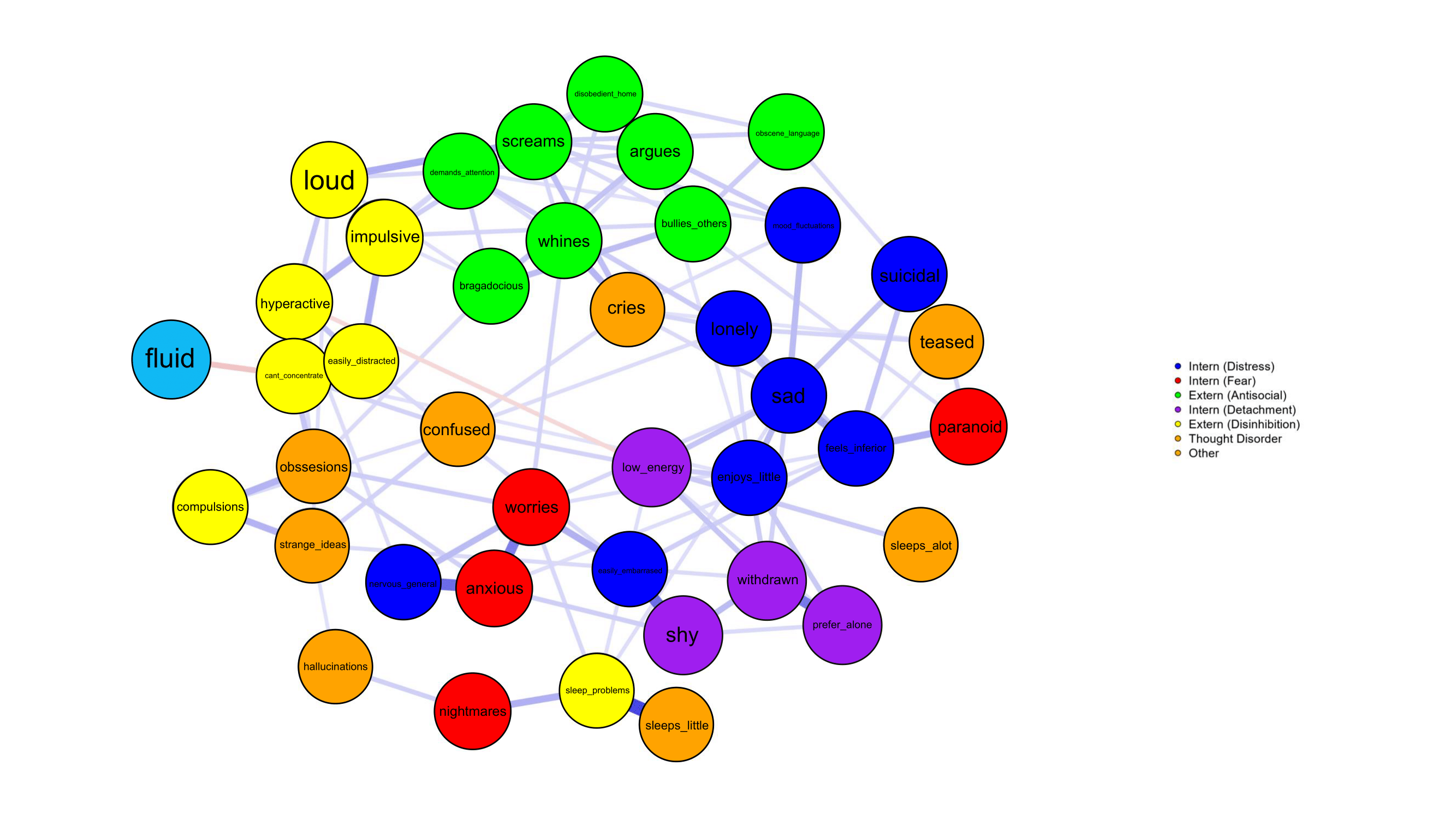


[Supplementary Figure S10: Partial Correlation Networks for psychopathological variables using parent ratings from the CBCL and Fluid Intelligence outcomes. Network indicates that among all psychopathological features, concentration problems have the highest association with fluid intelligence outcomes when controlling for all other variables.]


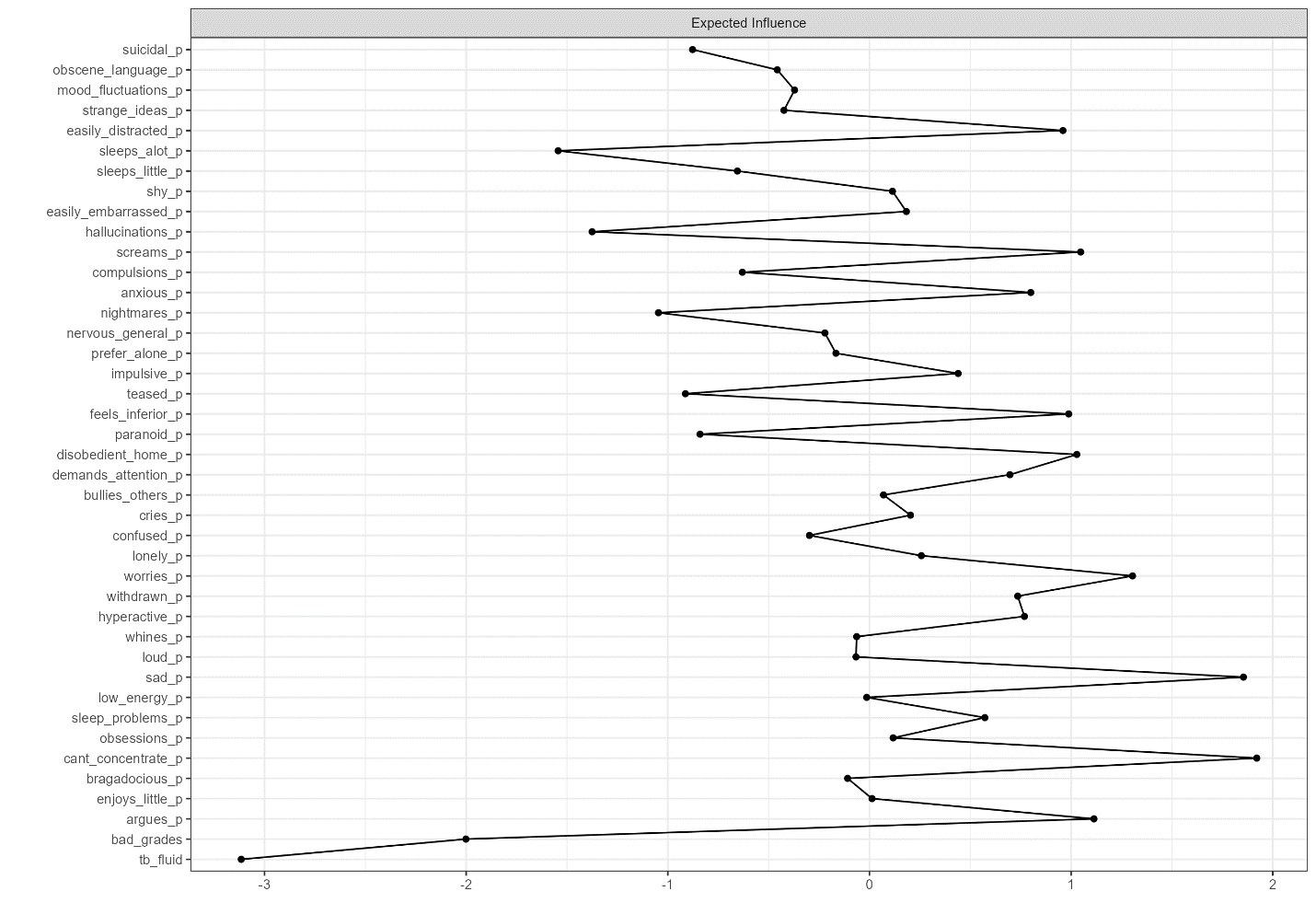


[Supplementary Figure S10b: Expected influence centrality metrics for larger-scale analysis of psychopathological features and fluid intelligence outcomes in ABCD cohort. The centrality of concentration problems appears to scale with wider range of psychopathological variables.]

## S11: DAG

[Supplementary Figure S11: DAG with a less conservative threshold (than shown above) with the direction of arrows that appear in at least 51% of the 500 bootstrapped networks. Similar to the other DAG, distractibility component of attention dysregulation similarly occurs as an upstream node indicating potential predictive priority.]

## S12: Half-Split Reliability Analyses

Half-Split Analyses for Correlational Analyses covered in Figure 1


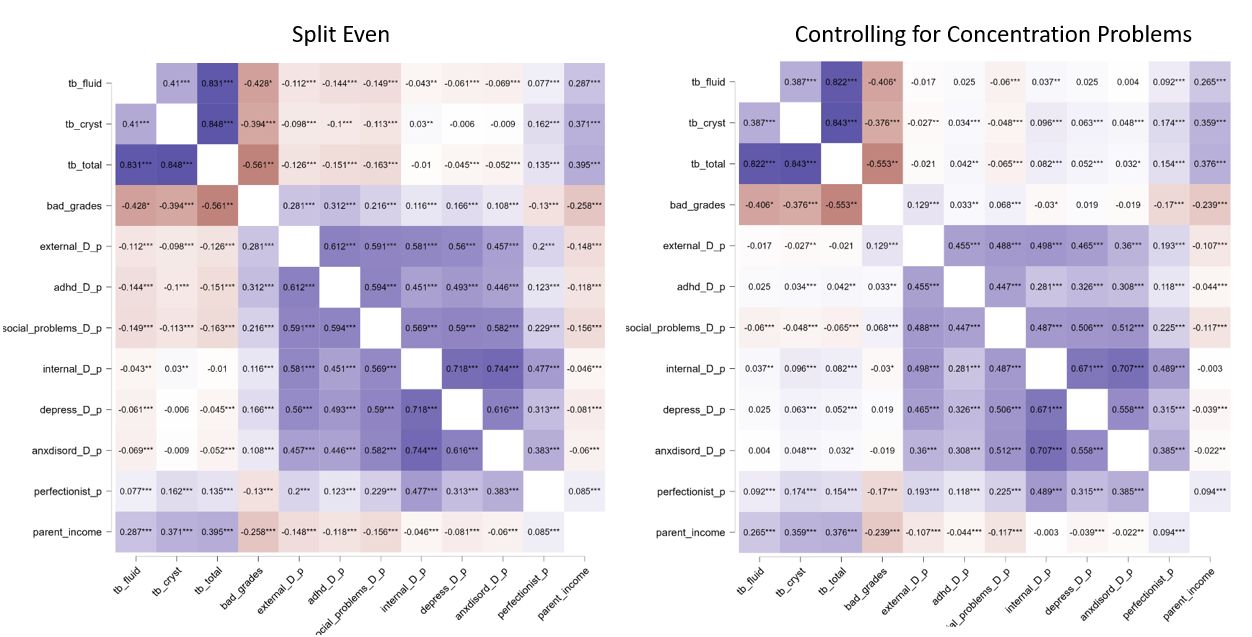


[Figure S12A: Split even analyses from figure 1.]


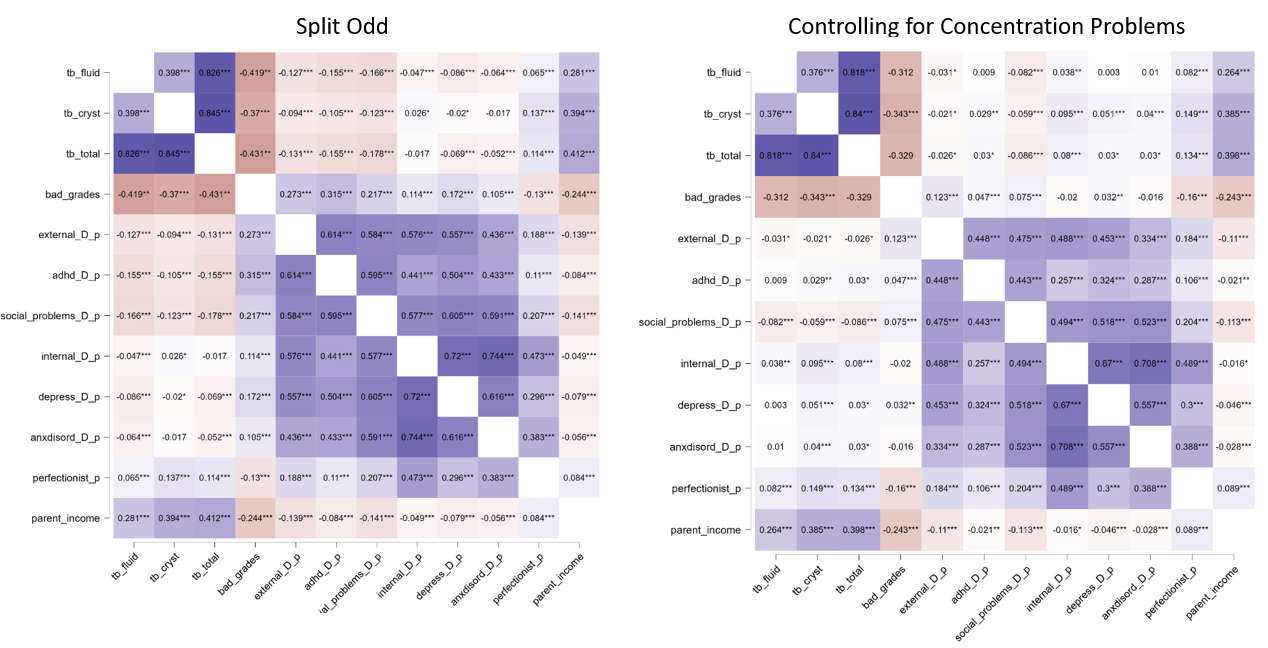


[Figure S12B: Split Odd analyses from figure 1.]

Half-Split Analyses for Stratification Analyses covered in Figures 2 & 3

| Half | Predictor | Cognitive performance type | Df | F | P-Value |
| --- | --- | --- | --- | --- | --- |
| Even | Depression | Fluid | 4, 4246 | 9.259 | <.001*** |
|  |  | Crystallized | 4, 7754 | 7.555 | <.001*** |
|  |  | Total | 4, 4253 | 8.877 | <.001*** |
|  | Anxiety | Fluid | 4, 4253 | 17.100 | <.001*** |
|  |  | Crystallized | 4, 4245 | 8.309 | <.001*** |
|  |  | Total | 9, 10,562 | 8.154 | <.001*** |
| Odd | Depression | Fluid | 4, 4253 | 19.557 | <.001*** |
|  |  | Crystallized | 4, 7509 | 11.557 | <.001*** |
|  |  | Total | 4, 4253 | 15.637 | <.001*** |
|  | Anxiety | Fluid | 4, 4246 | 9.147 | <.001*** |
|  |  | Crystallized | 4, 7554 | 7.902 | <.001*** |
|  |  | Total | 4, 4253 | 13.217 | <.001*** |

[Supplementary Table 12A. Odd-even split-half analysis of internal consistency reliability in depression/ADHD and anxiety/ADHD psychopathological stratifications on measures of cognitive performance. Similar to results using the full sample, those with higher levels of attention dysregulation in both subsamples show impairment in all measures of cognitive performance across depression and anxiety dimensions. (Half= half of sample; Predictor= psychopathological dimension [top= depression, bottom= anxiety]; Cognitive performance measure= type of cognitive performance [top= fluid, middle= crystallized, bottom= total]; Df= degrees of freedom; F= F-statistic; P-Value= significance level of test. *p < .05, **p < .01, ***p < .001.)]

| Half | Predictor | Cognitive performance type | Df | F | P-Value |
| --- | --- | --- | --- | --- | --- |
| Even | Parental income | Fluid | 9, 5229 | 22.383 | <.001*** |
|  |  | Crystallized | 9, 8689 | 76.686 | <.001*** |
|  |  | Total | 9, 5244 | 97.689 | <.001*** |
|  | Depression & anxiety | Fluid | N/A | N/A | >.05 |
|  |  | Crystallized | 4, 9479 | 4.888 | <.001*** |
|  |  | Total | 4, 5735 | 5.06 | <.001*** |
|  | Concentration difficulties | Fluid | 2, 5738 | 24.674 | <.001*** |
|  |  | Crystallized | 2, 9479 | 32.491 | <.001*** |
|  |  | Total | 2, 5375 | 32.397 | <.001*** |
| Odd | Parental income | Fluid | 9, 5271 | 24.862 | <.001*** |
|  |  | Crystallized | 9, 8688 | 91.876 | <.001*** |
|  |  | Total | 9, 5288 | 114.702 | <.001*** |
|  | Depression & anxiety | Fluid | N/A | N/A | >.05 |
|  |  | Crystallized | 4, 9485 | 2.837 | .023* |
|  |  | Total | 4, 5783 | 3.921 | .003** |
|  | Concentration difficulties | Fluid | 2, 5776 | 28.353 | <.001*** |
|  |  | Crystallized | 2, 9485 | 35.654 | <.001*** |
|  |  | Total | 2, 5783 | 154.987 | <.001*** |

[Supplementary Table 12B. Odd-even split-half analysis of internal consistency reliability of 2-way ANOVA results of parental income (top), depression and anxiety symptomatology (middle), and concentration difficulties (bottom) on types of cognitive performance. In both subsamples- similar to the full sample, parental income and concentration difficulties significantly influenced all three types of cognitive performance, while depression and anxiety influenced only crystallized and total cognitive performance (Half= half of sample; Predictor= psychopathological dimension [top= depression, bottom= anxiety]; Cognitive performance measure= type of cognitive performance [top= fluid, middle= crystallized, bottom= total]; Df= degrees of freedom; F= F-statistic; P-Value= significance level of test. *p < .05, **p < .01, ***p < .001.)]

## S13: Sample Sizes & Descriptive Statistics for Stratification Analyses

| **Descriptives - Fluid** | | | | | | | | | | | |
| --- | --- | --- | --- | --- | --- | --- | --- | --- | --- | --- | --- |
| **anxadhd_c** | | **N** | | **Mean** | | **SD** | | **SE** | | **Coefficient of variation** | |
| highanx_highadhd |  | 656 |  | 91.452 |  | 17.725 |  | 0.692 |  | 0.194 |  |
| highanx_lowadhd |  | 392 |  | 100.071 |  | 16.279 |  | 0.822 |  | 0.163 |  |
| lowanx_highadhd |  | 175 |  | 92.277 |  | 17.239 |  | 1.303 |  | 0.187 |  |
| lowanx_lowadhd |  | 2589 |  | 99.099 |  | 17.851 |  | 0.351 |  | 0.180 |  |
| other |  | 4640 |  | 95.847 |  | 16.906 |  | 0.248 |  | 0.176 |  |
|  | | | | | | | | | | | |

| **Descriptives - Crystalized** | | | | | | | | | | | |
| --- | --- | --- | --- | --- | --- | --- | --- | --- | --- | --- | --- |
| **anxadhd_c** | | **N** | | **Mean** | | **SD** | | **SE** | | **Coefficient of variation** | |
| highanx_highadhd |  | 660 |  | 102.753 |  | 18.482 |  | 0.719 |  | 0.180 |  |
| highanx_lowadhd |  | 393 |  | 112.653 |  | 18.855 |  | 0.951 |  | 0.167 |  |
| lowanx_highadhd |  | 176 |  | 105.511 |  | 18.979 |  | 1.431 |  | 0.180 |  |
| lowanx_lowadhd |  | 2598 |  | 108.038 |  | 18.654 |  | 0.366 |  | 0.173 |  |
| other |  | 4661 |  | 106.803 |  | 17.962 |  | 0.263 |  | 0.168 |  |
|  | | | | | | | | | | | |

| **Descriptives - Fluid** | | | | | | | | | | | |
| --- | --- | --- | --- | --- | --- | --- | --- | --- | --- | --- | --- |
| **depadhd_c** | | **N** | | **Mean** | | **SD** | | **SE** | | **Coefficient of variation** | |
| highdep_highadhd |  | 709 |  | 91.426 |  | 17.734 |  | 0.666 |  | 0.194 |  |
| highdep_lowadhd |  | 279 |  | 100.033 |  | 17.037 |  | 1.020 |  | 0.170 |  |
| lowdep_highadhd |  | 220 |  | 90.877 |  | 17.753 |  | 1.197 |  | 0.195 |  |
| lowdep_lowadhd |  | 3192 |  | 98.880 |  | 17.611 |  | 0.312 |  | 0.178 |  |
| other |  | 4052 |  | 95.833 |  | 16.814 |  | 0.264 |  | 0.175 |  |
|  | | | | | | | | | | | |

| **Descriptives - Crystalized** | | | | | | | | | | | |
| --- | --- | --- | --- | --- | --- | --- | --- | --- | --- | --- | --- |
| **depadhd_c** | | **N** | | **Mean** | | **SD** | | **SE** | | **Coefficient of variation** | |
| highdep_highadhd |  | 713 |  | 103.604 |  | 18.792 |  | 0.704 |  | 0.181 |  |
| highdep_lowadhd |  | 279 |  | 113.053 |  | 18.108 |  | 1.084 |  | 0.160 |  |
| lowdep_highadhd |  | 222 |  | 101.396 |  | 16.971 |  | 1.139 |  | 0.167 |  |
| lowdep_lowadhd |  | 3205 |  | 108.126 |  | 18.248 |  | 0.322 |  | 0.169 |  |
| other |  | 4069 |  | 106.829 |  | 18.283 |  | 0.287 |  | 0.171 |  |
|  | | | | | | | | | | | |

| **Descriptives – Total Cognitive Performance** | | | | | | | | | | | |
| --- | --- | --- | --- | --- | --- | --- | --- | --- | --- | --- | --- |
| **depanx_c** | | **N** | | **Mean** | | **SD** | | **SE** | | **Coefficient of variation** | |
| highdep_highanx |  | 820 |  | -0.066 |  | 1.019 |  | 0.036 |  | -15.470 |  |
| highdep_lowanx |  | 120 |  | 0.218 |  | 1.086 |  | 0.099 |  | 4.978 |  |
| lowdep_highanx |  | 220 |  | 0.065 |  | 0.939 |  | 0.063 |  | 14.434 |  |
| lowdep_lowanx |  | 2668 |  | 0.119 |  | 1.017 |  | 0.020 |  | 8.564 |  |
| other |  | 4623 |  | 0.098 |  | 0.980 |  | 0.014 |  | 10.021 |  |
|  | | | | | | | | | | | |

| **Descriptives – Total Cognitive Performance** | | | | | | | | | | | | | |
| --- | --- | --- | --- | --- | --- | --- | --- | --- | --- | --- | --- | --- | --- |
| **depanx_c** | | **Cant_Concentrate_p** | | **N** | | **Mean** | | **SD** | | **SE** | | **Coefficient of variation** | |
| highdep_highanx |  | 0 |  | 185 |  | 0.319 |  | 1.018 |  | 0.075 |  | 3.191 |  |
|  |  | 1 |  | 373 |  | -0.065 |  | 0.972 |  | 0.050 |  | -14.878 |  |
|  |  | 2 |  | 262 |  | -0.338 |  | 1.001 |  | 0.062 |  | -2.958 |  |
| highdep_lowanx |  | 0 |  | 54 |  | 0.450 |  | 1.010 |  | 0.137 |  | 2.245 |  |
|  |  | 1 |  | 51 |  | 0.084 |  | 1.019 |  | 0.143 |  | 12.141 |  |
|  |  | 2 |  | 15 |  | -0.158 |  | 1.428 |  | 0.369 |  | -9.023 |  |
| lowdep_highanx |  | 0 |  | 132 |  | 0.249 |  | 0.887 |  | 0.077 |  | 3.563 |  |
|  |  | 1 |  | 72 |  | -0.189 |  | 0.897 |  | 0.106 |  | -4.752 |  |
|  |  | 2 |  | 16 |  | -0.310 |  | 1.201 |  | 0.300 |  | -3.875 |  |
| lowdep_lowanx |  | 0 |  | 2183 |  | 0.195 |  | 1.022 |  | 0.022 |  | 5.230 |  |
|  |  | 1 |  | 455 |  | -0.204 |  | 0.907 |  | 0.043 |  | -4.452 |  |
|  |  | 2 |  | 30 |  | -0.572 |  | 1.026 |  | 0.187 |  | -1.793 |  |
| other |  | 0 |  | 2640 |  | 0.315 |  | 0.947 |  | 0.018 |  | 3.004 |  |
|  |  | 1 |  | 1635 |  | -0.151 |  | 0.948 |  | 0.023 |  | -6.278 |  |
|  |  | 2 |  | 348 |  | -0.383 |  | 0.925 |  | 0.050 |  | -2.416 |  |
|  | | | | | | | | | | | | | |

| **Descriptives – Total Cognitive Performance** | | | | | | | | | | | | | |
| --- | --- | --- | --- | --- | --- | --- | --- | --- | --- | --- | --- | --- | --- |
| **Concentration Problems** | | **Parent Income** | | **N** | | **Mean** | | **SD** | | **SE** | | **Coefficient of variation** | |
| 0 |  | 1 |  | 146 |  | -0.817 |  | 0.868 |  | 0.072 |  | -1.063 |  |
|  |  | 10 |  | 706 |  | 0.704 |  | 0.941 |  | 0.035 |  | 1.336 |  |
|  |  | 2 |  | 151 |  | -0.459 |  | 0.924 |  | 0.075 |  | -2.014 |  |
|  |  | 3 |  | 93 |  | -0.468 |  | 0.910 |  | 0.094 |  | -1.945 |  |
|  |  | 4 |  | 213 |  | -0.332 |  | 0.850 |  | 0.058 |  | -2.559 |  |
|  |  | 5 |  | 266 |  | -0.195 |  | 0.967 |  | 0.059 |  | -4.971 |  |
|  |  | 6 |  | 408 |  | 0.020 |  | 0.938 |  | 0.046 |  | 46.529 |  |
|  |  | 7 |  | 683 |  | 0.129 |  | 0.954 |  | 0.037 |  | 7.422 |  |
|  |  | 8 |  | 766 |  | 0.377 |  | 0.893 |  | 0.032 |  | 2.370 |  |
|  |  | 9 |  | 1762 |  | 0.481 |  | 0.888 |  | 0.021 |  | 1.846 |  |
| 1 |  | 1 |  | 111 |  | -1.113 |  | 0.805 |  | 0.076 |  | -0.723 |  |
|  |  | 10 |  | 277 |  | 0.348 |  | 0.848 |  | 0.051 |  | 2.437 |  |
|  |  | 2 |  | 115 |  | -0.765 |  | 0.864 |  | 0.081 |  | -1.130 |  |
|  |  | 3 |  | 74 |  | -0.794 |  | 0.765 |  | 0.089 |  | -0.964 |  |
|  |  | 4 |  | 137 |  | -0.553 |  | 0.858 |  | 0.073 |  | -1.550 |  |
|  |  | 5 |  | 174 |  | -0.502 |  | 0.859 |  | 0.065 |  | -1.711 |  |
|  |  | 6 |  | 221 |  | -0.423 |  | 0.894 |  | 0.060 |  | -2.115 |  |
|  |  | 7 |  | 410 |  | -0.116 |  | 0.911 |  | 0.045 |  | -7.888 |  |
|  |  | 8 |  | 357 |  | 0.021 |  | 0.899 |  | 0.048 |  | 43.091 |  |
|  |  | 9 |  | 710 |  | 0.137 |  | 0.845 |  | 0.032 |  | 6.183 |  |
| 2 |  | 1 |  | 40 |  | -1.120 |  | 0.817 |  | 0.129 |  | -0.730 |  |
|  |  | 10 |  | 33 |  | 0.207 |  | 1.042 |  | 0.181 |  | 5.029 |  |
|  |  | 2 |  | 41 |  | -0.828 |  | 0.884 |  | 0.138 |  | -1.067 |  |
|  |  | 3 |  | 29 |  | -0.878 |  | 0.797 |  | 0.148 |  | -0.908 |  |
|  |  | 4 |  | 50 |  | -0.853 |  | 0.804 |  | 0.114 |  | -0.942 |  |
|  |  | 5 |  | 55 |  | -0.773 |  | 0.784 |  | 0.106 |  | -1.013 |  |
|  |  | 6 |  | 76 |  | -0.524 |  | 0.951 |  | 0.109 |  | -1.814 |  |
|  |  | 7 |  | 92 |  | -0.268 |  | 0.909 |  | 0.095 |  | -3.394 |  |
|  |  | 8 |  | 88 |  | -0.019 |  | 0.928 |  | 0.099 |  | -49.094 |  |
|  |  | 9 |  | 167 |  | 0.014 |  | 0.919 |  | 0.071 |  | 64.558 |  |
|  | | | | | | | | | | | | | |

[Supplementary Tables 13. Sample sizes and descriptive statistics for stratification analyses presented in Figures 2 & 3 in main manuscript.]
